# A systematic literature review to evaluate the cardiac and cerebrovascular outcomes of patients with Fabry disease treated with agalsidase Beta

**DOI:** 10.3389/fcvm.2024.1415547

**Published:** 2025-01-21

**Authors:** Gavin Y. Oudit, Pronabesh DasMahapatra, Nicole Lyn, Florence R. Wilson, Adekemi Adeyemi, Chae Sung Lee, Ana Crespo, Mehdi Namdar

**Affiliations:** ^1^ Division of Cardiology, Department of Medicine, Faculty of Medicine and Dentistry, University of Alberta, Edmonton, AB, Canada; ^2^ Heart Function Clinic, Mazankowski Alberta Heart Institute, University of Alberta, Edmonton, AB, Canada; ^3^ Specialty Care, Sanofi, Cambridge, MA, United States; ^4^ PRECISIONheor, Vancouver, BC, Canada; ^5^ Specialty Care, Sanofi, Milan, Italy; ^6^ Cardiology Division, Geneva University Hospitals, Geneva, Switzerland

**Keywords:** Fabry disease, agalsidase beta, genetic disorders, cardiac, cardiovascular, enzyme replacement therapy

## Abstract

**Background:**

Agalsidase beta is used to treat Fabry disease (FD); however, data on cardiac and cerebrovascular outcomes with agalsidase beta treatment come from studies with limited numbers of patients.

**Methods:**

A systematic literature review of studies reporting on the efficacy and effectiveness of agalsidase beta in FD was conducted. Studies were identified in searches of MEDLINE, Embase, and the Cochrane Central Register of Controlled Trials from January 2000–June 2022. Outcomes of interest included cardiac structure and mass, cardiac events, and cerebrovascular events.

**Results:**

Fifty-two citations (41 studies) were included. Reductions in interventricular septal thickness (IVST) and/or left ventricular posterior wall thickness (LVPWT) were demonstrated in six studies (follow-up 1–6 years, *n* = 4 using echocardiography, *n* = 2 cardiac MRI). IVST ranged from 12.1–14.9 mm at baseline and 10.8–14.1 mm at follow-up (all *p* < 0.05). LVPWT ranged from 11.7–16.0 mm at baseline and 10.7–13.0 mm at follow-up (all *p* < 0.05). Significant reductions in cardiac mass were demonstrated after 1 year of treatment in a single-arm study using cardiac MRI [left ventricular mass (LVM) 193–178 g; LVM index 102–94 g/m^2^; both *p* < 0.05]. Rates of composite cardiac events (3.8%–24.0%; four studies, follow-up 2–10 years) and cerebrovascular events (0.0%–18.9%; 12 studies, follow-up 1–10 years) were numerically lower than rates for placebo (follow-up 3 years).

**Conclusion:**

Literature over the last 20 years indicates that agalsidase beta treatment may lead to stabilization or regression of cardiac structural thickness and mass, and reduction in cardiac and cerebrovascular events relative to placebo.

## 1 Introduction

Fabry disease (FD) is a rare, X-linked genetic disorder caused by a deficiency in the activity of the lysosomal enzyme alfa-galactosidase A (α-gal A) (1). Over 1,000 pathogenic variants in the α-gal A gene (*GLA*) have been identified that interfere with the normal function of the α-gal A enzyme, resulting in the progressive lysosomal accumulation of globotriaosylceramide (GL3) and related sphingolipids in various types of cells including endothelial cells, podocytes, smooth muscle cells, cardiomyocytes, and neurons (1, 2). The deposition of GL3 in various organs increases the risk of irreversible damage and renal, cardiac, cardiovascular, and cerebrovascular clinical events, which can lead to death (3, 4).

Phenotypically, FD can be divided into classic or later-onset (non-classic) disease (5, 6). Males with the classic phenotype demonstrate symptoms typically manifesting in childhood, and have markedly reduced α-gal A enzyme activity, resulting in the accumulation of GL3 in the vascular endothelial cells of the heart, kidneys, brain, and skin (6–8). Comparatively, males diagnosed with the later-onset phenotype have relatively higher residual activity of α-gal A and usually present with a milder form of the disease at a later stage (6). In females, the clinical manifestations of FD range from asymptomatic to severe classic disease depending on the type of genetic mutation and the extent of X-chromosome inactivation (Lyonization), with skewed Lyonization significantly impacting phenotype (6, 9, 10).

Cardiac or cardiovascular involvement, which is often evident on electrocardiogram (11–13), typically begins early in patients with FD, involving the accumulation of GL3 in multiple cell types, including cells of the conduction system, cardiomyocytes, endothelial cells, and vascular smooth muscle cells (14). The resulting progressive cardiac damage can manifest as diastolic dysfunction, left ventricular hypertrophy (LVH), reduced myocardial blood flow, microvascular ischemia, fibrosis, arrhythmias, and conduction disturbances (2). These changes serve as indicators of cardiac involvement and measures of cardiac function and outcomes in FD (14). Eventually, the resulting GL3 accumulation and cascade of pathophysiological changes may cause clinical events such as arrhythmias requiring defibrillator and/or pacemaker therapy, syncope, myocardial infarction (MI), heart failure, and sudden cardiac death (2, 4). These serious cardiac manifestations are a leading cause of premature death among patients and one of the main determinants of their prognosis (3, 15). Approximately 50.0% of deaths among males and females with FD are accounted for by cardiovascular manifestations (15). Cerebrovascular complications due to the accumulation of GL3 within the endothelium of intracranial blood vessels are also a major cause of morbidity (16, 17), with stroke and transient ischemic attacks (TIAs) being the most prevalent cerebrovascular events in FD (16–18).

Current therapeutic approaches for the treatment of FD include the reduction of accumulated GL3 through enzyme replacement therapy (ERT) or oral chaperone therapy, along with symptomatic and palliative treatments when needed (5). There are currently three approved ERTs available in a number of countries worldwide: agalsidase beta (Fabrazyme®; Sanofi), pegunigalsidase alfa (Elfabrio®; Chiesi) and agalsidase alfa (Replagal®; Takeda). Agalsidase alfa is currently approved in Europe and is marketed globally including the UK, Australia, Germany, China and Japan, among others (19). However, agalsidase beta (also approved in Europe and Canada, among others) and pegunigalsidase alfa (also approved in Europe, Northern Ireland, and UK, among others) are the only two approved ERTs in the United States, where the former is indicated for patients aged 2 years and older and the latter for adult patients only (19–24). The chaperone therapy migalastat (Galafold®; Amicus Therapeutics) was first conditionally approved in 2018 in the United States and Canada for use in adult patients with amenable variants (25, 26).

Given the rarity of FD, it is challenging to conduct clinical studies with large numbers of patients; consequently, sample sizes are limited in publications on the cardiovascular and cerebrovascular outcomes associated with the treatment of FD. The aim of this systematic literature review (SLR) was to evaluate the clinical efficacy and effectiveness of agalsidase beta on cardiovascular and cerebrovascular outcomes in patients with FD from a collection of all such studies over 20 years.

## 2 Methods

An SLR was conducted to identify studies on the clinical efficacy and effectiveness, and immunogenicity of agalsidase beta in patients with FD. This paper focuses on cardiac and cerebrovascular outcomes from the search.

## 3 Study identification and selection

Relevant studies were identified by searching MEDLINE, Embase, and the Cochrane Central Register of Controlled Trials (CCTR). Database searches were performed using the Ovid platform. The initial searches were conducted for the period January 2000 to March 2019 with pre-defined search strategies (Supplementary Tables S1–S3). A supplementary search was conducted in May 2019 in which the search strings “exp enzyme replacement/” (Embase), “exp Enzyme Replacement Therapy/” (MEDLINE and CCTR), and “ERT.mp” (all three databases) were added to ensure that all agalsidase beta studies were captured, including those that did not use “Fabrazyme” or “agalsidase beta” as index terms. An update to the SLR was performed in June 2022 for the period March 2019 to June 2022 using the same pre-defined search strategies (Supplementary Tables S4–S6). The database searches were augmented with hand searches of the bibliographies of key studies of interest, including recently published SLRs and meta-analyses.

Study eligibility criteria were defined in terms of the population, interventions, comparators, outcomes, and study design structure outlined in Table 1. These criteria guided the identification and selection of studies relevant for the SLR. The population search terms were adapted from previously published SLRs and included both adult and pediatric study populations with FD (27–29). The intervention of interest was agalsidase beta; however, natural history and placebo could be selected as comparators. Agalsidase alfa or switch data were included if no other data were available. Outcomes of interest presented in this paper included cardiac structure and mass, as well as cardiac and cerebrovascular events (Table 1). Eligible studies included clinical trials and prospective or retrospective observational studies; case reports of a single patient and narrative review articles were excluded. Only full-text publications in English language were included, and therefore conference abstracts and presentations were excluded from the evidence base.

**Table 1:** Population, interventions, comparators, outcomes, and study design criteria for study selection.

Criteria	Description
Population	Patients of any age with FD
Interventions	Agalsidase beta (Fabrazyme®)
Comparators	• Placebo • Natural history • Agalsidase alfa (Replagal®) (*included as a comparator only if there were no other comparator data or switch data available*)
Outcomes^a^	• Cardiac morphology and mass: ○ LVH ○ LVM/LVMI ○ LVPWT ○ IVST • Cardiac events: ○ Symptomatic arrhythmia ○ Angina ○ MI ○ Cardiovascular devices (pacemaker, defibrillator) • Cardiac events as a composite outcome • CNS/cerebrovascular events: ○ Stroke ○ TIA ○ WML • Renal markers: ○ GFR – measured or estimated ○ Proteinuria/UPCR ○ Serum creatinine level/change • Renal events: ○ Renal events as a composite outcome ○ Chronic dialysis ○ Renal transplantation ○ ESRD • All-cause mortality • Gastrointestinal symptoms • Disease severity: ○ MSSI ○ DS3 • Quality of life: ○ SF-36 ○ EuroQoL • Pain: ○ BPI ○ McGill pain questionnaire • Immunogenicity: ○ Anti-drug antibodies ○ Neutralizing antibodies ○ IgG antibodies ○ Serum-mediated inhibition
Study design	• Clinical trials, including randomized controlled trials, non-randomized controlled trials, and single-arm trials • Observational studies (prospective and retrospective); except case reports

BPI, brief pain inventory; CNS, central nervous system; DS3, Disease Severity Scoring System; ESRD, end-stage renal disease; FD, Fabry disease; GFR, glomerular filtration rate; IgG, immunoglobulin G; IVST, interventricular septal thickness; LVH, left ventricular hypertrophy; LVM, left ventricular mass; LVMI, left ventricular mass index; LVPWT, left ventricular posterior wall thickness; MI, myocardial infarction; MSSI, Mainz Severity Score Index; SF-36, Short Form-36; SLR, systematic literature review; TIA, transient ischemic attack; UPCR, urinary albumin-urinary creatinine ratio; and WML, white matter lesion.

^a^ All outcomes were selected as part of the SLR; this manuscript describes only cardiovascular (cardiac morphology and mass, cardiac events, and cardiac events as a composite outcome) and cerebrovascular outcomes (CNS/cerebrovascular events).

This SLR was conducted and reported in accordance with the Preferred Reporting Items for Systematic Reviews and Meta-Analyses (PRISMA) statement (30, 31). Two independent reviewers reviewed all abstracts and proceedings identified by the search according to the selection criteria; however, the outcome criteria were only applied during the screening of full-text publications. All eligible studies identified during abstract screening were then evaluated at full-text stage by two independent reviewers, and a third reviewer was included to reach consensus for any conflicts. The full-text studies identified as eligible were included for data extraction.

### 3.1 Data extraction

Data were extracted from all eligible studies on study characteristics, interventions, patient characteristics, and outcomes. In cases where there were multiple publications from a single study, data were extracted from the publication with the most complete dataset; however, some data were extracted from multiple publications to capture outcomes at different follow-up times or for different subgroups.

### 3.2 Study quality assessment

Study quality was assessed by two independent reviewers. Following reconciliation between the two reviewers, a third reviewer was included to reach consensus for any remaining discrepancies. The risk of bias in randomized clinical trials was assessed using the Cochrane Collaboration's Risk of Bias tool (Supplementary Table S7) (32). The quality of observational studies and non-randomized clinical trials was assessed using the Newcastle–Ottawa Scale (Supplementary Tables S8 and S9) (33).

## 4 Results

**Figure 1:**
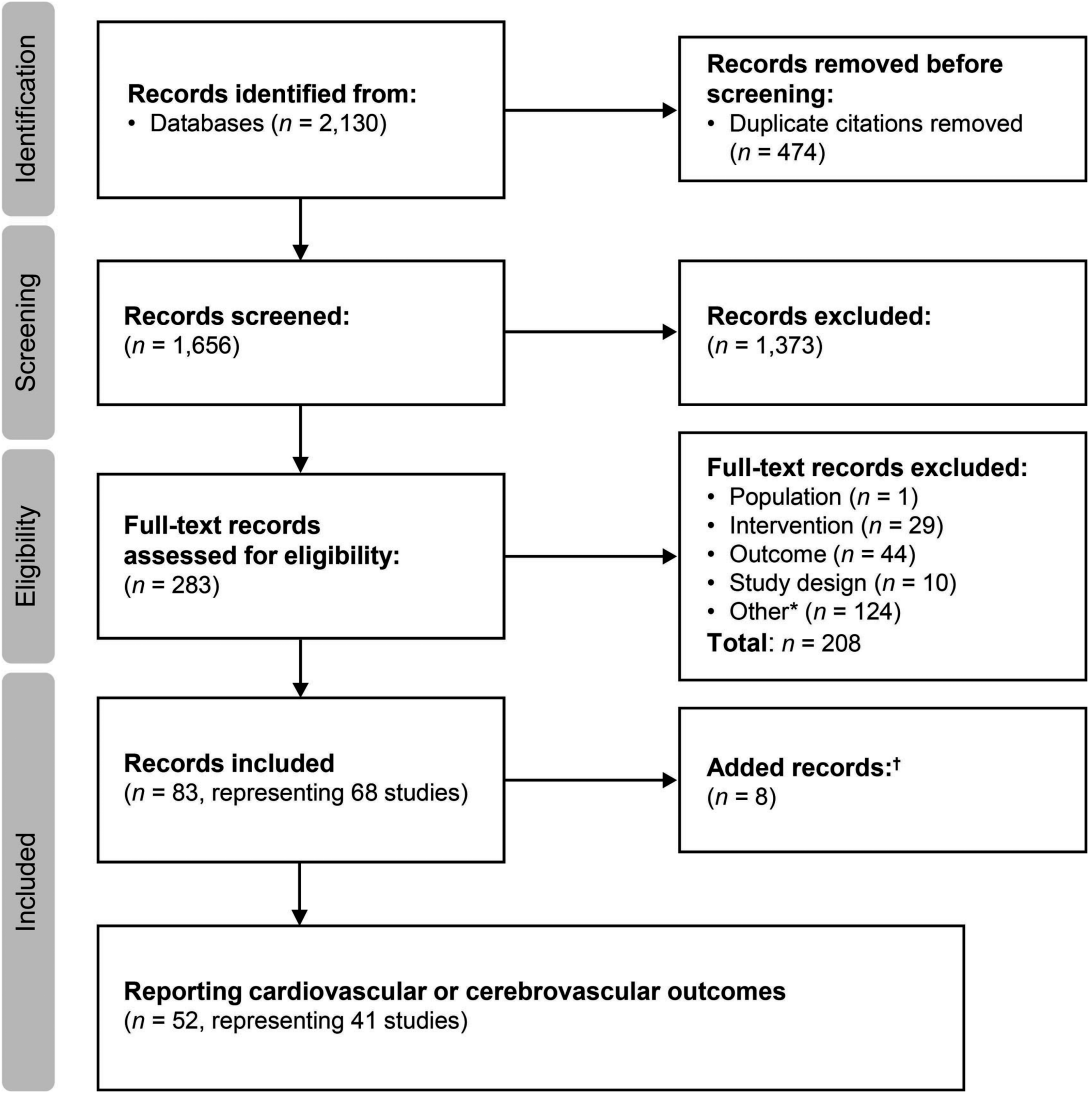
Study selection flow diagram (2000–2022). ^a^Includes conference abstracts and duplicate records; ^b^additional studies identified through hand searches of key study references, or supplemental searches.

Searches were conducted on March 13, 2019 and June 15, 2022 for the period from January 2000 to June 2022. The flow diagram for the study selection process is presented in Figure 1. A total of 83 citations corresponding to 68 studies were eligible for inclusion in the SLR. Of these, 52 citations corresponding to 41 studies reported cardiovascular and/or cerebrovascular outcomes. These comprised nine interventional studies [including four randomized controlled trials (RCTs)] and 32 observational studies (Table 1). Key characteristics of the included studies are summarized in Supplementary Table S10. Most studies were assessed to be of good quality with a low risk of bias (Supplementary Tables S11–S13). There was limited reporting of cardiovascular and cerebrovascular outcome data in five studies (four citations, one article included the results of two studies) (34–37) and one article (38) reported the pooled outcomes of two RCTs; as such, these five citations were not included in the below results. Therefore, cardiovascular and cerebrovascular outcomes were reviewed from 47 citations (36 studies). A summary of baseline patient characteristics for the selected studies is presented in Table 2.

**Table 2:** Summary of baseline patient characteristics.

Study	Adult or pediatric	Fabry phenotype^a^	*N*	Age, years		Male, *n* (%)	Treatment naïve, *n* (%)	Renal function	
				Mean (SD)	Median (range)			eGFR	Proteinuria
Interventional studies									
Banikazemi et al., 2007 (NCT00074984) (39)	Adult	Classic	51	46.9 (9.8)	NR	45 (88)	51 (100)	Mean (SD) ml/min/1.73 m^2^: 53 (17.7)	Mean (SD) UPCR, g/g: 1.5 (1.5)
Germain et al., 2015 (NCT00074971; NCT00196742) (40)	Adult (>16 years)	Classic	52	30.1 (10.0)	29 (17–62)	50 (96)	52 (100)	Mean (SD) ml/min/1.73 m^2^ Low renal involvement (*n* = 32): 126.0 (18.71) High renal involvement (*n* = 20): 101.6 (22.78)	Mean (SD) UPCR, g/g Low renal involvement (*n* = 32): 0.2 (0.1) High renal involvement (*n* = 20): 1.3 (0.9)
Ramaswami et al., 2019 (NCT00701415) (41)	Pediatric	Classic (except 1 patient with later-onset)	31	11.6 (4.2)	12 (5–18)	31 (100)	31 (100)	Mean (SD) ml/min/1.73 m^2^ iGFR: 118.9 (18.8) eGFR: 101.4 (17.3)	Mean (SD) mg/g UACR 11.0 (6.0) UPCR 93.6 (36.3)
Kalliokoski et al., 2006 (42)	Adult	Unclear	10	34 (11)	(19–49)	7 (70)	10 (100)	NR	NR
Spinelli et al., 2004 (43)	Adult	Later-onset	9	40.9	32 (25–61)	7 (78)	9 (100)	NR	NR
Tahir et al., 2007 (NCT00343577) (44)	Adult	Classic/unknown (*n* = 10); not reported (*n* = 1)	11^b^	NR	37.1 (18.3–56.7)	8 (73)	11 (100)	NR	NR
Vedder et al., 2007 (ISRCTN45178534) (45)	Adult	Unclear	34	Agalsidase beta group 48^c^ (14) Agalsidase alfa group 42^c^ (13)	Agalsidase beta group 47 (24–76) Agalsidase alfa group 44 (19–60)	Agalsidase beta group 9 (56) Agalsidase alfa group 9 (50)	34 (100)	Median (SD) GFR, ml/min Agalsidase beta group: 108 (31) Agalsidase alfa group: 99 (33)	Median (range) g/24 h Agalsidase beta group: 0.24 (0.10–0.68) Agalsidase alfa group: 0.25 (0.06–2.65)
Wraith et al., 2008 (NCT00074958) (46)	Pediatric	Classic	16	12.1 (2.5)	11.7 (8.5–16.0)	14 (88)	16 (100)	Mean (SD) GFR ml/min/1.73 m^2^: 126 (29)	Mean (SD) mg/m^2^/24 h: 118 (55)
Wuest et al., 2011 (47)	Adult	Unclear	14	42 (8)	(32–55)	11 (79)	14 (100)	Mean (SD) GFR, ml/min: 83 (32)	NR
Observational studies: Fabry Disease Registry (NCT00196742)									
Germain et al., 2013 (48)	Adult	Unclear^d^	115	37.5 (10.4)	37 (19–74)	115 (100)	115 (100)	Mean (SD) ml/min/1.73 m^2^: 79 (42)	Mean (SD) UPCR, g/g: 1.0 (1.66)
Hopkin et al., 2016 (49)	Adult and pediatric	Classic or unknown	1,411	Males: 35 (14.2) Females: 44 (14.3)	NR	969 (69)	1,411 (100)	NR	NR
Wanner et al., 2020 (50)	Adult	Classic or unknown	LVH: 42 eGFR: 86	LVH: 51.1 (10.7) eGFR: 47.2 (11.5)	LVH: 50.0 (25th–75th percentile 44.2–59.1) eGFR: 46.3 (25th–75th percentile 38.8–56.2)	0 (0)	42/86 (100)	Slope: −0.83 ml/min/1.73 m^2^/year (95% CI:1.52, −0.13) during the pre-treatment period	NR
Other observational studies									
Beer et al., 2006 (51)	Adult	Severe (likely classic)	17	41 (8)	NR	17 (100)	NR	NR	NR
Breunig et al., 2006 (52)	Adult	Unclear	26	41.4 (8.9)	(29–57)	20 (77)	26 (100)	Mean (SD) GFR, ml/min/1.73 m^2^ GFR <90 at baseline: 71 (17) GFR >90 at baseline: 115 (18)	Mean (SD) mg/day GFR <90 at baseline (*n* = 4): 2091 (743) GFR >90 at baseline (*n* = 6): 845 (714)
Collin et al., 2011 (53)	Adult	Classic	46	Treated 34.9 (12.3); untreated 31.6 (12.7)	NR	Treated 28 (93); untreated 15 (94)	46 (100)	Mean (SD) ml/min/m^2^ Treated: 108 (51) Untreated: 103 (52)	NR
Elliott et al., 2006 (54)	Adult	Classic	10	53.8 (10.9)	(43–82)	10 (100)	10 (100)	NR	NR
Imbriaco et al., 2009 (Source: Messalli et al., 2012) (55, 56)	Adult	Unclear^e^	16	39 (12)	NR	10 (63)	16 (100)	NR	NR
Koskenvuo et al., 2008 (57)	Adult	Unclear	9	41.4	(20–65)	5 (56)	9 (100)^f^	Mean (SD) GFR, ml/min: 114 (30)	NR
Lenders et al., 2020 (58)	Adult	Classic (*n* = 57); non-classic (mild and later-onset; *n* = 5)	62	Agalsidase alfa: naïve (*n* = 4) 56 ± 16; long-term (*n* = 32) 48 ± 16 Agalsidase beta: naïve (*n* = 11) 46 ± 17; long-term (*n* = 15) 51 ± 14		34 (55)	15 (24)	Mean (SD) ml/min/1.73 m^2^ Agalsidase alfa: naïve (*n* = 4) 69.5 (24.4); long-term (*n* = 32) 83.2 (38.4) Agalsidase beta: naïve (*n* = 11) 96.7 (24.5); long-term (*n* = 15) 63.8 (30.0)	Median (range) ACR, mg/g: Agalsidase alfa: naïve (*n* = 4) 76 (22–220); long-term (*n* = 32) 111 (10–2,810) Agalsidase beta: naïve (*n* = 11) 177 (23–2,436); long-term (*n* = 15) 175 (15–4,773)
Pisani et al., 2005 (59)	Adult	Classic	9	45.3	47 (26–60)	8 (89)	9 (100)	NR	NR
Politei et al., 2014 (60)	Adult (*n* = 5); pediatric (*n* = 1; aged 17 years)	Classic	6	NR	(17–50)	4 (67)	6 (100)	NR	NR
Tsurumi et al., 2021 (NCT00233870; AGAL02904) (35)^g^	Adult and pediatric	Classic (56.0%) and later-onset	307	(*n* = 307) 39.5 (95% CI: 37.7, 41.3)	NR	201 (65.5)	NR	Renal impairment was present in 37.5% of patients at baseline	NR
Arends et al., 2018 (61)	Adult	Classic (*n* = 278); later-onset (*n* = 109)	387	46 (15)	NR	Agalsidase beta group: 78 (56); Agalsidase alfa group: 116 (47)	387 (100)	Median (range) ml/min/1.73 m^2^ Agalsidase beta group: 86 (10–140) Agalsidase alfa group: 89 (10–159)	NR
Kim et al., 2016 (62)	Adult (*n* = 15); pediatric (*n* = 4)	Classic (*n* = 18) unknown (*n* = 1)	19	31.6	35.5 (8.4–48.1)	15 (79)	19 (100)	Mean (SD) ml/min/1.73 m^2^ Female: 115.9 (28.1) Male: 107.3 (28.5)	Mean (SD) mg/day Female: 72.3 (17.2) Male: 734.4 (792)^c^
Motwani et al., 2012 (63)	Adult	Unclear	66	42 (14)	NR	44 (67)	66 (100)	NR	NR
Riccio et al., 2021 (34)	Adult	Classic (*n* = 38); later-onset (*n* = 15)	53	46.75 (14.44)	NR	28 (53)	42 (79)	NR	NR
Rombach et al., 2012 (64)	Adult	Classic	59^h^	NR	Males: (17.0–65.3) Females: 45.6 (15.9–71.5)	29 (49)	59 (100)	NR	NR
Tsuboi, 2007 (65)	Adult	Classic and later-onset	11	50.1	53 (28–67)	5 (45)	11 (100)	NR	NR
Siamopoulos, 2004 (36)	Adult	Classic^i^	2	50.5	(50–51)	2 (100)	2 (100)	NR	NR
Koeppe et al., 2012 (66)	Adult	Unclear	25	40 (9)	(18–55)	21 (84)	NR	NR	NR
Machann et al., 2011 (47)	Adult	Unclear	8	43 (8)	NR	8 (100)	8 (100)	NR	NR
Observational switch studies: CFDI registry									
Lenders et al., 2021 (67)	Adult	Classic (except two patients)	78	44 (13)	NR	49 (63)	NR	Mean (SD) ml/min/1.73 m^2^ Re-switch: 80 (29) Switch: 102 (30) Agalsidase beta regular dose: 103 (24)	NR
Other observational switch studies									
Limgala et al., 2019 (37)	Adult and pediatric	Classic (except three patients with later-onset)	27	31 (17)	(8–59)	12 (44)	NR	NR	NR
Politei et al., 2016 (68)	Adult	Classic	12	Agalsidase beta group 31.5^c^; agalsidase alfa group 32^c^; Agalsidase beta re-switch 33.5^c^	NR	Agalsidase beta group 6 (75); agalsidase alfa group 2 (50); Agalsidase beta re-switch 2 (100)	12 (100)	NR	NR
Lin et al., 2014 (69)	Adult (*n* = 8); pediatric (*n* = 1)	Classic (*n* = 3); later-onset (*n* = 6)	9	43.3^c^	42.8^c^ (14.4–66.7)	9 (100)	9 (100)	NR	NR
Ripeau et al., 2017 (70)	Adult and pediatric	Unclear	33	32.4 (2.0)	Males: (10.0–51.5) Females: (25.1–55.9)	23 (70)	0 (0)	Mean (SD) ml/min/1.73 m^2^: 106.1 (5.1)	Mean (SD) UprotV: 708.2 (259.5) mg/day UPCR: 0.66 (0.27) mg/g
Vedder et al., 2008 (71)	Adult	Classic, later-onset, and unclear^j^	52	NR	Agalsidase beta 1.0 mg/kg: 48 (27–70); Agalsidase beta 0.2 mg/kg: 49 (25–73); Agalsidase alfa: 47 (19–62)	Agalsidase beta 1.0 mg/kg 10 (48); Agalsidase beta 2.0 mg/kg 8 (62); Agalsidase alfa 10 (56)	52 (100)	Median (range) CrCl, ml/min Agalsidase beta 1.0 mg/kg: 98 (43–142); Agalsidase beta 0.2 mg/kg: 108 (53–154); Agalsidase alfa: 101 (22–136)	NR
Pisani et al., 2013 (72)	Adult	Classic	10	44 (5)	(38–56)	7 (70)	10 (100)	Mean (SD) ml/min/1.73 m^2^: 92.4 (13.1)	Mean (SD) UPCR, mg/mMol: 0.96 (0.29)
Tsuboi & Yamamoto, 2012 (73)	Adult	Classic and later-onset	11	47.3 (14.3)	NR	4 (36.4)	0 (0)	Mean (SD) ml/min/1.73 m^2^: 89.97 (21.51)	NR
Weidemann et al., 2014 (74)	Adult	Classic, later-onset, and unclear	105	45.3 (12.8)	NR	62 (59.1)	0 (0)	Median (range) ml/min/1.73 m^2^: 99 (63–119)^k^	Median (range) ACR, mg/g: median 205 (0–1010)^k^
van der Veen, 2022 (75)	Adult and pediatric	Classic	7^l^	NR	24 (14–26)	7 (100)	0 (0)	Median (range) ml/min/1.73 m^2^: 116 (92–132)	Median (range) UACR mg/mMol: 0.4 (0–8.8)

Data were extracted from publications with the most complete dataset.

α-gal A, alpha galactosidase A; ACR, albumin–creatinine ratio; CFDI, Canadian Fabry Disease Initiative; CrCl, creatinine clearance; eGFR, estimated glomerular filtration rate; GFR, glomerular filtration rate; iGFR, insulin-like glomerular filtration rate; NR, not reported; SD, standard deviation; UACR, urine albumin–creatinine ratio; UPCR, urine protein–creatinine ratio; and UprotV, urine protein excretion rate.

^a^ Unknown phenotype: mutation details were not reported in the publication as stated by the authors; unclear phenotype: mutation details were not mentioned by the authors and cannot be inferred based on limited information available.

^b^ Includes two patients from Banikazemi et al., 2007 (39).

^c^ Calculated.

^d^ Plasma α-gal A activity (nmol/hour/ml) was available in 25 patients, and ranged from 0 to 18.

^e^ Median baseline α-gal A enzymatic activity in males was 0.25 nmol/h/ml, and values ranged from 0.2–4.2 nmol/h/ml (normal range 4.0–21.9); enzyme activity at baseline in the three females ranged from 2.1–4.2 nmol/h/ml.

^f^ All patients had received ERT continuously from 2003 but underwent a 3-month cessation in the therapy before the first evaluation in this study in 2004.

^g^ Pooled results from two studies.

^h^ Includes some patients from Vedder et al., 2007 (45).

^i^ Both patients had T385P mutation, which is classic in phenotype; plasma α-gal A activity was 0.8 and 0.7 nmol/h/ml, respectively.

^j^ Male phenotypes: 12 classic, 2 later-onset, 14 mutations unknown.

^k^ Average of agalsidase beta regular dose group, agalsidase beta dose-reduction group, and agalsidase alfa switch group.

^l^ Includes seven patients from Ramaswami et al., 2019 (41), with 10-year follow-up.

### 4.1 Cardiac parameters

A total of 36 publications corresponding to 29 studies reported cardiac structure [interventricular septal (IVST) and left ventricular posterior wall thickness (LVPWT)] and cardiac mass [left ventricular mass (LVM) or LVM index (LVMI)] parameters, including six interventional studies which are summarized in Tables 3, 4. Data from all 29 studies are summarized in Supplementary Tables S14 and S15. Most available data on cardiac structure and mass outcomes were from studies in adults.

**Table 3:** IVST and LVPWT in interventional studies of patients with FD treated with agalsidase beta.

Study	Treatment (follow-up duration)	Imaging modality	*N*	Mean (SD) IVST (mm)			Mean (SD) LVPWT (mm)		
				Baseline value	Follow-up value	*p* value^a^	Baseline value	Follow-up value	*p* value**^a^**
Germain et al., 2015 (NCT00074971; NCT00196742) (40)	Agalsidase beta 1.0 mg/kg EOW (120 months)	Echo	50^b^	10.7 (2.3)	11.5 (3.1)	NR	10.6 (2.3)	11.7 (3.0)	NR
Kalliokoski et al., 2006 (42)	Agalsidase beta 1.0 mg/kg EOW (12 months)	Echo	7	Systolic: 15.7 (0.9)	Systolic: 17.3 (1.7)	0.19	Systolic: 17.7 (1.9)	Systolic: 18.0 (1.8)	0.69
				Diastolic: 10.9 (1.6)	Diastolic: 12.1 (2.1)	0.25	Diastolic: 10.3 (1.1)	Diastolic: 10.0 (1.0)	0.63
Spinelli et al., 2004 (43)	Agalsidase beta 1.0 mg/kg EOW (12 months)	Echo	9	14 (3)^c^	13.3 (2.7)^c^ *At 6 months of follow-up*	<0.025	13.3 (1.6)	13 (2.5) *At 6 months of follow-up*	>0.05
					12.4 (1.2)^c^ *At 12 months of follow-up*			12.4 (1.5) *At 12 months of follow-up*	
Ramaswami et al., 2019 (NCT00701415) (41)	Agalsidase beta 0.5 mg/kg EOW (5 years)	Echo	16^d^	NR	NR	NR	8 (1) [*n* = 22]	8 (1) [*n* = 21]	0.2814 (Changes unremarkable)
	Agalsidase beta 1.0 mg/kg q4w (5 years)		15^d^	NR	NR	NR			

Data were extracted from publications with the most complete dataset.

Echo, echocardiography; EOW, every other week; ERT, enzyme replacement therapy; IVST, interventricular septum thickness; LVPWT, left ventricular posterior wall thickness; q4w, every four weeks; NR, not reported; and SD, standard deviation.

^a^ p values refer to comparison between follow-up and baseline values, unless otherwise stated.

^b^ Population was a mix of adult and pediatric patients.

^c^ Calculated from Figure 1 in Spinelli et al. 2004 (43).

^d^ Pediatric patients.

**Table 4:** LVM and LVMI in interventional studies of patients with FD treated with agalsidase beta.

Study	Treatment (follow-up duration)	Imaging modality	*N*	Mean (SD) LV mass (g)			Mean (SD) LVMI (g/m^2^)		
				Baseline value	Follow-up value	*p* value^a^	Baseline value	Follow-up value	*p* value^a^
Kalliokoski et al., 2006 (42)	Agalsidase beta 1.0 mg/kg EOW (12 months)	Echo	7	275 (28)	292 (35)	0.27	151 (12)	156 (14)	0.37
Spinelli et al., 2004 (43)	Agalsidase beta 1.0 mg/kg EOW (12 months)	Echo	9	Significant decrease in LV mass (*p* < 0.001)			183 (44.5)	174.5 (41.7)^b^ *At 6 months of follow-up*	NR
								169.5 (37.2)^b^ *At 12 months of follow-up*	<0.01
Vedder et al., 2007 (ISRCTN45178534) (45)	Agalsidase beta 0.2 or 1.0 mg/kg EOW (24 months)	Echo	16	Median (SD): 313 (91)	296 (76) *At 12 months of follow-up*	NS^c^	NR		
					308 (90) *At 24 months of follow-up*				
	Agalsidase alfa 0.2 mg/kg EOW (24 months)		18	Median (SD): 279 (101)	244 (89) *At 12 months of follow-up*				
					294 (87) *At 24 months of follow-up*				
Wuest et al., 2011 (47)	Agalsidase beta 1.0 mg/kg EOW (13 months)	Cardiac MRI	14	193 (47)	178 (51)	<0.05	102 (26)	94 (27)	<0.05
Ramaswami et al., 2019 (NCT00701415) (41)	Agalsidase beta 0.5 mg/kg EOW (5 years)	Echo	16^d^	NR	NR	NR	33.2 (5.1) g/m^2.7^ [*n* = 23]	31.4 (7.2) g/m^2.7^ [*n* = 24]	Changes unremarkable
	Agalsidase beta 1.0 mg/kg q4w (5 years)		15^d^						

Data were extracted from publications with the most complete dataset.

Echo, echocardiography; EOW, every other week; ERT, enzyme replacement therapy; LV, left ventricle; LVH, left ventricular hypertrophy; LVM, left ventricular mass; LVMI, left ventricle mass index; NS, not significant; q4w, every four weeks; MRI, magnetic resonance imaging; NR, not reported; and SD, standard deviation.

^a^ *p* values refer to comparison between follow-up and baseline values, unless otherwise stated.

^b^ Calculated from Figure 1 in Spinelli et al., 2004 (43).

^c^ After 12 and 24 months of treatment no reduction in LV mass was seen, which was not different between the two treatment groups.

^d^ Pediatric patients.

In adult patients, IVST and LVPWT was reported in 22 publications, and LVM or LVMI were reported in 32 publications (Supplementary Tables S14 and S15). Overall, most patients treated with agalsidase beta showed stabilization or improvement in cardiac structure or cardiac mass parameters, demonstrated by non-significant changes or reductions from study baseline in IVST, LVPWT, and LVM or LVMI.

### 4.2 Cardiac structure (IVST and LVPWT)

Four interventional studies investigated IVST or LVPWT in patients treated with agalsidase beta (Table 3) (40–43). In a single-arm study of nine patients in Italy with later-onset disease, IVST was significantly reduced from 14.0 mm at baseline to 12.4 mm after 12 months of follow-up (*p* < 0.025) (43). A numerical reduction in LVPWT from 13.3 mm to 12.4 mm was also demonstrated; however, this change was not statistically significant (43). Three further interventional studies reported stabilization of cardiac wall thickness after treatment with agalsidase beta, with no significant changes observed between baseline and follow-up (range 1–10 years) (40–42). These included a 10-year follow-up of 50 patients (classic phenotype) from the Fabry Registry who had participated in the phase 3 pivotal trial for agalsidase beta and its open-label extension; non-significant changes from baseline to 10 years in IVST (10.7–11.5 mm) and LVPWT (10.6–11.7 mm) were demonstrated, with both measures remaining within the normal range (40). Patients with cardiomyopathy in these studies presented with a range of hypertrophic phenotypes and baseline characteristics, with individual variation in the response to ERT but an overall improvement in LV mass and LV stiffness (40–42).

A total of 13 observational studies investigated the effects of agalsidase beta on IVST and LVPWT, and these generally showed consistent results with the interventional studies (Supplementary Table S14) (50, 52, 55, 56, 58–60, 66, 76, 77). Of note, significant reductions in both IVST (13.5–11.9 mm; *p* < 0.0001) and LVPWT (13.2–11.4 mm; *p* < 0.0001) were reported at 72-month follow-up in a prospective cohort study in Germany (77). Whereas data from the Fabry Registry demonstrated that LVPWT and IVST stabilized during 3.6 years of treatment in female patients treated with agalsidase beta, regardless of renal involvement or antiproteinuric agent use (50). Several other observational studies also reported significant improvements or non-significant increases in IVST and/or LVPWT in patients receiving agalsidase beta (Supplementary Table S14) (55, 56, 58–60, 66), regardless of sex (58). For example, an open-label study assessing the long-term effects of agalsidase beta also reported significantly reduced LVPWT after 48 months of treatment (16.0 mm vs. 13.0 mm; *p* < 0.001) (55).

### 4.3 Cardiac mass (LVM/LVMI)

Five interventional studies investigated cardiac mass in patients treated with agalsidase beta (Table 4) (41–43, 45, 47). In one study, significant reductions from baseline in cardiac mass were demonstrated in a cohort of nine patients with FD (43). At 12 months of follow-up, LVMI (calculated from echocardiographs using the Penn convention) was significantly reduced from 183 g/m^2^ to 169.5 g/m^2^ (*p* < 0.01) (43). A single-arm study of 14 patients in Germany also demonstrated significant reductions in LVM and LVMI [determined based on cardiac magnetic resonance imaging (MRI)] at 13 months (LVM: 193.0–178.0 g; LVMI: 102–94 g/m^2^; both *p* < 0.05) (47). A further two interventional studies reported no significant changes in cardiac mass at 12 or 24 months of follow-up (42, 45). These included an RCT of 34 patients treated with either agalsidase beta or agalsidase alfa in which there was no significant reduction in LVM (determined by echocardiography) from baseline to 24 months, and no significant difference between treatments (agalsidase beta: median 313–308 g; agalsidase alfa: 279–294 g) (45).

Seven observational studies demonstrated significant improvements in cardiac mass from baseline with agalsidase beta treatment (Supplementary Table S15) (47, 51, 53, 55, 56, 63, 66, 76, 77), regardless of sex (63). In a retrospective cohort study of 387 patients with classic or later-onset disease, there was no significant difference in LVMI (calculated from echocardiographs using the Devereux formula) between those treated with agalsidase beta compared with agalsidase alfa over the first year of treatment (*p* = 0.15) (61). However, a higher proportion of patients treated with agalsidase beta had a reduction in LVMI (79.0%) at 1 year compared with patients treated with agalsidase alfa [62.0%; odds ratio (OR): 2.27, 95% confidence interval (CI): 1.11, 4.86; *p* = 0.03] (61). The proportion of males in the agalsidase alfa (47%) and agalsidase beta (56%) groups may confound these results and should be interpreted with caution due to the observational nature of the study, since female patients generally have less LVH, may have different clinical presentations and response patterns to treatment with ERT (61). A prospective cohort study of nine patients also showed that after 24 months of therapy with agalsidase beta, progression of LVMI decreased in all but two patients (59). Prior to initiation of agalsidase beta, the average percentage increase per year in LVMI was 6.0%, which decreased to 3.0% during the 3 years of treatment (59). Another study assessed the natural progression of LVH in untreated male patients with FD and the left ventricular changes after 2 years of treatment of agalsidase beta (48). The results showed that LVM progressively increased in untreated males; however, males who began treatment with agalsidase beta at an earlier age experienced significant decline in LVM compared with untreated males (mean LVM slope for patients who initiated treatment before the age of 30 was −3.6 g/year compared with +9.5 g/year in untreated males within the same age bracket) (48).

### 4.4 Cardiac parameters according to disease severity at baseline

Several studies demonstrated differences in cardiac outcomes with agalsidase beta depending on disease severity at baseline (Supplementary Tables S14 and S15) (51, 52, 63, 66, 76). For example, an observational study reported reduction in LVPWT at 24-month follow-up, but significance was only reached in the subgroup of patients with normal renal function (estimated glomerular filtration rate >90 ml/min/1.73 m^2^; *p* = 0.017) (52). Three studies were identified in which patients were classified based on the presence of myocardial fibrosis visualized using MRI with late enhancement (LE) (51, 66, 76). Two studies showed significant improvements in IVST and LVPWT or cardiac mass in patients without cardiac fibrosis at baseline after 12–36 months of agalsidase beta treatment (51, 76). In these studies, improvements in IVST and LVPWT or cardiac mass were also observed in patients with mild or severe fibrosis, or LE-positive MRI; however, these changes were not statistically significant (51, 76). A further prospective case-control study demonstrated that LVM was significantly higher in patients who were LE positive compared with LE negative at baseline (*p* < 0.05) (66). After 12 months of agalsidase beta treatment, mean LVM decreased in LE positive and LE negative patients; however, the difference between baseline and follow-up values was not statistically significant (66). A retrospective study of registry data from England reported that in 42 patients with LVH at baseline, LVMI was significantly reduced by agalsidase beta; however, there was no change for those patients without baseline LVH (63).

### 4.5 Cardiac parameters in pediatric patients with FD

Three studies were identified that reported follow-up data for cardiac structural outcomes in pediatric patients treated with agalsidase beta (Supplementary Tables S14 and S15) (41, 62, 75). An open-label study of 31 males aged 5–18 years treated with 0.5 mg/kg EOW or 1.0 mg/kg every 4 weeks (q4w) of agalsidase beta demonstrated unremarkable changes in wall thickness and LVMI after 5 years (41). Cardiac mass (LVMI determined by echocardiogram) was reported to remain within normal range after 5–10 years of treatment for four male South Korean patients (aged 8–17 years) in one retrospective cohort study (62). In addition, significantly lower LVM was observed in a cohort of seven males with classic disease who had been treated with agalsidase beta for approximately 10 years since childhood, compared with untreated controls (median 80 g/m^2^ vs*.* 94 g/m^2^ by echocardiography; median 53 g/m^2^ vs*.* 68 g/m^2^ by cardiac MRI; both *p* = 0.02) (75).

### 4.6 Cardiac events

A total of 17 publications from 14 studies reported cardiac events (Tables 4, 5; Supplementary Table S16).

**Table 5:** Composite cardiac events reported between baseline and follow-up in patients with FD treated with agalsidase beta.

Study	Definition of composite event	Treatment (follow-up duration)	*N*	Patients with ≥1 event, *n* (%)	Comparative measure
Interventional studies					
Banikazemi et al., 2007 (NCT00074984) (39)	MI; new symptomatic arrhythmia requiring antiarrhythmic medication, pacemaker, direct current cardioversion, or defibrillator implantation; unstable angina defined by national practice guidelines and accompanied by electrocardiographic changes resulting in hospitalization; or worsening congestive heart failure requiring hospitalization	Agalsidase beta 1.0 mg/kg EOW (35 months)	51	3 (5.9)	OR: 0.42 (95%: CI 0.058, 2.7), *p* = 0.42^a^
		Placebo (35 months)	31	4 (12.9)	
Germain et al., 2015 (NCT00074971; NCT00196742) (40)	Cardiac-related death and MI	Agalsidase beta 1.0 mg/kg EOW (120 months)	52^b^	2 (3.8)^c^	NR
Observational studies: Fabry Disease Registry					
Hopkin et al., 2016 (49)	Cardiac-related death, MI, first-time congestive heart failure, AF, ventricular tachycardia, evidence of progressive heart disease severe enough to require pacemaker placement, bypass surgery, coronary artery dilatation, or implantation of a cardioverter defibrillator	Agalsidase beta 1.0 mg/kg EOW (38–52 months)^d^	1,411^b^	116 (8.2)^e^	NR
Other observational studies					
Breunig et al., 2006 (52)	MI with subsequent coronary intervention, coronary artery bypass grafting, cardiac pacemaker implantation, and new onset of AF	Agalsidase beta 1.0 mg/kg EOW (23 months)	25	6 (24.0)	NR

Data were extracted from publications with the most complete dataset. Benichou et al., 2009 (78) reports the pooled outcomes of Banikazemi et al., 2007 (39) and Germain et al., 2007 (79) clinical trials, and was therefore not included in this table.

AF, atrial fibrillation; CI, confidence interval; EOW, every other week; ERT, enzyme-replacement therapy; MI, myocardial infarction; OR, odds ratio; and NR, not reported.

^a^ Adjusted for baseline imbalance in proteinuria; unadjusted and adjusted values were the same; *p* value refers to comparison between agalsidase beta and placebo.

^b^ Population was a mix of adult and pediatric patients.

^c^ Two cardiac events were reported: cardiac-related death in patient with low renal involvement at age 52 and MI in patient with high renal involvement at age 53.

^d^ Median treatment follow-up was 4.3 years in males and 3.2 years in females.

^e^ On-ERT events were cardiac in 85 males (9.0% of all males) and in 31 females (7.0% of all females).

### 4.7 Cardiac events as a composite outcome

Two interventional studies and two observational studies reported composite cardiac events in adult patients treated with agalsidase beta, using varying definitions of composite events (Table 5) (39, 40, 49, 52). Across studies, the incidence of composite cardiac events in agalsidase beta-treated patients ranged from 3.8%–24.0%. Data from the phase 3 pivotal trial for agalsidase beta showed that patients treated with agalsidase beta experienced fewer composite cardiac events over 35 months of follow-up than patients treated with placebo [5.9% vs*.* 12.9%; OR (95% CI): 0.42 (0.058, 2.7)], although the comparison was not statistically significant (*p* = 0.42) (39). The 10-year follow-up results from the phase 3 study of agalsidase beta showed that the majority (50/52; 96.2%) of patients with classic FD did not experience any cardiac events; only one patient experienced cardiac-related death and one patient experienced an MI (40). In a large prospective cohort study of the Fabry Registry, over 90.0% of patients did not experience a cardiac event while on agalsidase beta treatment over 3 to 4 years of follow-up; however, compared with other events, cardiac events in this study were more common than non-cardiac events (49). The proportion of male patients who experienced cardiac events (9.0%) was slightly greater than in female patients (7.0%) (49). Of note, patients who experienced cardiac events while on agalsidase beta treatment were more likely to have experienced a cardiac event pre-treatment than those without cardiac events (49).

### 4.8 Individual cardiac events

Data on individual cardiac events (angina, MI, and pacemaker/defibrillator implantation events) were reported in eight studies in adults (Supplementary Table S16). Two interventional studies with follow-up of ≥12 months to 35 months reported angina events in adult patients; however, neither study described how angina was defined or measured (39, 42). In both studies, no events of angina were reported in patients treated with regular-dose agalsidase beta (*n* = 51 and *n* = 10) (39, 42); however, one angina event was reported in a placebo arm (*n* = 31) (39). Low rates of MI (1.9%–4.0%) were reported in three studies, with only one agalsidase beta-treated patient in each study experiencing an MI event (39, 40, 52). In the 10-year follow-up of the phase 3 study of agalsidase beta, one of 52 patients with classic FD (1.9%) treated with agalsidase beta experienced an MI (40). The incidence of pacemaker and/or defibrillator implantation events in adults treated with agalsidase beta was reported in six studies; across all study designs and follow-up durations (ranging from 12–88 months), the incidence of events was 0.0%–27.3% (37, 44, 52, 65, 67, 77, 80). Two studies reporting cardiac events in pediatric patients reported normal cardiac function during treatment with agalsidase beta (46, 62).

The incidence of arrhythmias, atrial fibrillation (AF), and other electrocardiogram (ECG) abnormalities in adult patients treated with agalsidase beta was reported in seven studies (follow-up ≥12 months to 8.1 years) and ranged from 3.9%–66.7% (Table 6) (39, 44, 45, 52, 62, 67, 75, 81). Of these, four studies evaluated the new onset of AF in adult patients (incidence of 5.6%–12.5% over 23–36 months follow-up) (Table 6) (44, 45, 52, 73). In a randomized open-label study, new-onset AF in patients receiving ERT for more than 24 months was reported in two patients treated with agalsidase beta (*n* = 2/16; after 42 and 36 months) and one patient treated with agalsidase alfa (*n* = 1/18; after 30 months) (45). While in a placebo-controlled phase 3 pivotal trial for agalsidase beta with over 35 months of follow-up, a higher proportion of patients treated with placebo experienced events of new-onset symptomatic arrhythmia requiring medication compared with patients treated with agalsidase beta (9.7% vs*.* 3.9%) (39). Data from a long-term prospective cohort study with median follow-up of 6 years, showed new-onset ventricular tachycardia in 30.0% of patients treated with agalsidase beta, but the event rate was not reported for the natural history control group (77). In this study, almost all patients with documented ventricular tachycardia had LVH with myocardial fibrosis, and all patients in the agalsidase beta group who died because of cardiac arrest had ventricular tachycardia (77). Other cardiac arrhythmias were detected in 66.7% of adult patients prior to initiating agalsidase beta and persisted throughout treatment in a small retrospective observational study in South Korea (*n* = 15 adults; mean follow-up 8 years; Table 6).

**Table 6:** Arrhythmia and AF events reported between baseline and follow-up in patients with FD treated with agalsidase beta.

Study		Definition	Treatment (follow-up duration)	*N*	Patients with ≥1 event, *n* (%)
Interventional studies					
Banikazemi et al., 2007 (NCT00074984) (39)		New symptomatic arrhythmia requiring antiarrhythmic medication	Agalsidase beta 1.0 mg/kg EOW (35 months)	51	2 (3.9)
			Placebo (35 months)	31	3 (9.7)
Tahir et al., 2007 (NCT00343577) (44)		New onset of AF	Agalsidase beta 1.0 mg/kg EOW (35 months)	11	1 (9.1)^a^
Vedder et al., 2007 (ISRCTN45178534) (45)		New onset of AF	Agalsidase beta 1.0 mg/kg EOW (24 months)	16	2 (12.5)
			Agalsidase alfa 0.2 mg/kg EOW (>24 months)	18	1 (5.6)
Observational studies					
Breunig et al., 2006	Breunig et al., 2006 (52)	New onset of AF	Agalsidase beta 1.0 mg/kg EOW (23 months)	25	2 (8.0)
	Weidemann et al., 2013 (77)	New ventricular tachycardia (for malignant arrhythmias)	Agalsidase beta 1.0 mg/kg EOW (72 months)	40	12 (30.0)
			Natural history (45 years)	40	NR
Kim et al., 2016 (62)		Cardiac arrhythmias, including bradyarrhythmia, Wolff–Parkinson–White syndrome, atrioventricular block, and bundle branch block	Agalsidase beta 1.0 mg/kg EOW, with some reduction due to shortage (mean: 8.1 years)	15^b^	Cardiac arrhythmias were observed in 9 adult males and 1 adult female at agalsidase beta initiation [10 (66.7)]. These conduction abnormalities persisted throughout agalsidase beta treatment.
Observational switch studies					
Tsuboi & Yamamoto, 2012 (73) [Source: Tsuboi & Yamamoto, 2014 (81)]		New onset of AF	Agalsidase beta 1.0 mg/kg EOW (24 months)	11	1 (9.1)^c^
			Agalsidase alfa 0.2 mg/kg EOW (36 months) *Following switch from agalsidase beta*		
Lenders et al., 2021 (67)		ECG abnormalities	Regular dose group (1.0 mg/kg (>12 months)	16	Baseline: 4 (25.0) Follow-up: 5 (31.3) RR: 1.17
			Switch group [beta to alfa (0.2 mg/kg)]	19	Baseline: 9 (25.0) Follow-up: 17 (43.6) RR: 1.01
			Re-switch group (beta to alfa for 12 months)	36	Baseline: 7 (36.8) Follow-up: 10 (52.6) RR: 1.39

Data were extracted from publications with the most complete dataset. Benichou et al., 2009 (78) reports the pooled outcomes of Banikazemi et al., 2007 (39) and Germain et al., 2007 (79) clinical trials, and was therefore not included in this table.

AF, atrial fibrillation; ECG, electrocardiogram; EOW, every other week; NR, not reported; and RR, relative risk.

^a^ One patient developed AF 5 months after completion of the observation period (i.e., 40 months after starting agalsidase beta), but it is unclear whether this patient was still receiving agalsidase beta at the time of AF event.

^b^ Population was a mix of adult and pediatric patients.

^c^ After switching from agalsidase beta to agalsidase alfa, one patient with classic FD developed AF, which resolved spontaneously within ∼6 months; it is not known whether the onset of the arrhythmia was associated with the natural course of FD or was triggered by the switch in medication.

Limited data were available on arrhythmias and AF events in pediatric patients receiving ERT with agalsidase beta. An observational study of seven male pediatric patients with classic disease after 10 years of treatment with agalsidase beta at a lower than standard dose (0.5 mg/kg EOW or 1.0 mg/kg q4w) did not demonstrate incomplete right bundle branch block in five patients with available data and sinus bradycardia in 14.3%; corresponding rates for the untreated comparison group were 31.3% and 52.4%, respectively (75). Low rates of sinus arrhythmia or conduction abnormalities were observed in an interventional study in 16 pediatric patients (0.0% at 48 weeks of follow-up) (46); similarly all pediatric patients (*n* = 4) showed normal cardiac function during treatment with agalsidase beta in a retrospective cohort study (5–10 year follow-up) (62).

### 4.9 Cerebrovascular ischemic events

A total of 18 publications based on 12 studies reported cerebrovascular ischemia events in patients treated with agalsidase beta. Across these studies, the rate of cerebrovascular ischemia events (defined as hemorrhagic or ischemic stroke, or TIA) in adult patients ranged from 0%–18.9% over follow-up periods from ≥12 months to 10 years (Table 7). In comparative studies, the rate of cerebrovascular ischemia events with agalsidase beta was similar or lower than the control group (Table 7) (39, 77, 82). The 35 months of follow-up results from the phase 3 pivotal trial for agalsidase beta showed that 6.5% of the patients in the placebo group experienced a cerebrovascular event compared with 0.0% of the patients in the agalsidase beta group [OR (95% CI): 0.00 (0.00, 3.20); *p* = 0.14] (39). In addition, in an observational study of 40 patients with FD in Germany, a numerically lower rate of stroke events in patients treated with agalsidase beta (10.0%; 72 months of follow-up duration) was reported compared with the natural history of disease (25.0%; 45 years of follow-up duration) (77). In one study of 16 pediatric patients, no stroke or TIA events were reported after 12 months of agalsidase beta treatment (46).

**Table 7:** Cerebrovascular ischemia events reported between baseline and follow in patients with FD treated with agalsidase beta.

Study		Definition	Treatment (follow-up duration)	*N*	Patients with ≥1 event, *n* (%)	Comparative measures	*p* value^a^
Interventional studies							
NCT00074984	Fellgiebel et al., 2014 (82)	Cerebral infarctions (stroke) were identified as present or absent on each brain image	Agalsidase beta 1.0 mg/kg EOW (27 months)	25	1 (4.0)	NR	NR
			Placebo (27 months)	16	2 (12.5)		
	Banikazemi et al., 2007 (39)	Defined as a stroke or TIA documented by a physician	Agalsidase beta 1.0 mg/kg EOW (35 months)	51	0 (0.0)^b^	OR: 0.00, 95% CI: 0.00, 3.20^c^	0.14
			Placebo (35 months)	31	2 (6.5)^d^		
Germain et al., 2015 (NCT00074971; NCT00196742) (40)		Symptomatic stroke or TIA	Agalsidase beta 0.5 mg/kg EOW (120 months)	52^e^	5 (9.6)^f^	NR	NR
Tahir et al., 2007 (NCT00343577) (44)		Cerebrovascular accident (stroke)	Agalsidase beta 1.0 mg/kg EOW (35.2 months)	11	0 (0.0)	NR	NR
Observational studies: FD Registry							
Hopkin et al., 2016 (49)		Cerebrovascular events: hemorrhagic or ischemic stroke, or stroke of unknown type	Agalsidase beta 1.0 mg/kg EOW (3.2–4.3 years)	1,411^e^	61 (4.3)	NR	NR
Other observational studies							
Arends et al., 2018 (61)		Cerebral events: stroke or TIA diagnosed by a neurologist	Agalsidase beta 1.0 mg/kg EOW or agalsidase alfa 0.2 mg/kg EOW (59 months)	387	25 (6.5)^g^	NR	NR
Breunig et al., 2006 (52) (Source: Weidemann et al., 2013) (77)		Any new onset of TIA	Agalsidase beta 1.0 mg/kg EOW (72 months)	40	3 (7.5)	NR	NR
			Natural history (45 years)	40	NR	NR	NR
		Stroke	Agalsidase beta 1.0 mg/kg EOW (72 months)	40	4 (10.0)^h^	NR	NR
			Natural history (45 years)	40	10 (25.0)^h^	NR	NR
Koskenvuo et al., 2008 (57)		Stroke	Agalsidase beta 1.0 mg/kg EOW (12 months)	9	1 (11.1)	NR	NR
Pisani et al., 2005 (59)		New cerebrovascular ischemic episode	Agalsidase beta 1.0 mg/kg EOW (24 months)	9	0 (0.0)	NR	NR
Observational switch studies							
Tsuboi & Yamamoto, 2012 (81) (Source: Tsuboi & Yamamoto 2014) (81)		Stroke	Agalsidase alfa 0.2 mg/kg EOW (12 months)	13	No patient suffered a stroke during the 12 months of follow-up after switching to agalsidase alfa.		NR
Weidemann et al., 2014 (74) (Source: Kramer et al., 2018) (80)		New stroke/TIA	Regular dose group Agalsidase beta 1.0 mg/kg EOW (≥12 months)	37	4 (10.8)^i^	RR: 0.20, 95% CI: 0.03, 0.29	<0.05
			Switch group Agalsidase beta to agalsidase alfa 0.2 mg/kg EOW (≥12 months)	38	2 (5.3)^i^	RR: 0.17, 95% CI: 0.01, 0.42	<0.05
			Re-switch group Agalsidase alfa to agalsidase beta 1.0 mg/kg EOW (≥12 months)	37	7 (18.9)^i^	RR: 1.00, 95% CI: 0.13, 7.50	NR
Lenders et al., 2021 (67)		Stroke/TIA	Regular dose group Agalsidase beta 1.0 mg/kg (>12 months) (88 ± 25 months)	17	2 (10.6)^j^	RR: 1.00, 95% CI: 0.35, 2.84	NR
			Switch group Reduced dose of agalsidase beta and subsequent switch to agalsidase alfa (0.2 mg/kg) (88 ± 25 months)	22	0^j^	NR	NR
			Re-switch group Re-switched to agalsidase beta after receiving agalsidase alfa for 12 months (88 ± 25 months)	39	5 (12.8)^j^	RR: 1.00, 95% CI: 0.51, 1.94	NR

Data were extracted from publications with the most complete dataset. Benichou et al., 2009 (78) reports the pooled outcomes of Banikazemi et al., 2007 (39) and Germain et al., 2007 (79) clinical trials, and was therefore not included in this table. Weidemann et al., 2014 (74) and Lenders et al., 2016 (83) reported data from shorter follow-up durations (one year and two years of follow-up, respectively) for the same study compared to Kramer et al., 2018 (80) (four years of follow-up), and therefore were not included in the table.

CI, confidence interval; EOW, every other week; HRI, high renal involvement; LRI, low renal involvement; NR, not reported; OR, odds ratio; RR, relative risk; and TIA, transient ischemic attack.

^a^ *p* values refer to comparison between follow-up and baseline values, unless otherwise stated.

^b^ No patients developed stroke or TIA.

^c^ Adjusted for baseline imbalance in proteinuria; unadjusted and adjusted values were the same.

^d^ Two patients developed stroke, none developed TIA.

^e^ Population was a mix of adult and pediatric patients.

^f^ The most frequent severe clinical event was stroke; ﬁve patients (9.6%; four LRI, one HRI) had a total of eight strokes.

^g^ Cerebral events (*n* = 25); causes of death in these patients included stroke (*n* = 1).

^h^ Prior to death, one patient had a stroke and another had a stroke prior to end-stage renal disease.

^i^ Calculated as the number of cumulative events between baseline and second long-term follow-up.

^j^ Events at 88 months follow-up for regular dose group, 44 months for switch group, and 53 months for re-switch group.

Five publications corresponding to four studies investigated white matter lesions [WMLs] burden over follow-up periods of ≥12 months to 12 years (Table 8) (41, 64, 68, 75, 82). There was no clear evidence to suggest a treatment benefit of either agalsidase beta or agalsidase alfa on WML burden. One placebo-controlled study showed a slight worsening in WML burden over 27 months in both agalsidase beta and placebo groups (mean increases of 0.7 and 1.0, respectively; difference not significant) (82). In another observational switch study a worsening of WML burden was reported in four patients, and one patient developed a new lesion during the 12 years of follow-up on treatment with either agalsidase beta or agalsidase alfa (68). Two studies of agalsidase beta were identified that reported on WML in pediatric patients (41, 75). In a RCT of 31 male pediatric patients, WMLs of unknown etiology were reported in one patient and remained stable over the 5-year follow-up; no new WML development was observed in any patient (41). In the subsequent retrospective cross-sectional study with 10-year follow-up, new WML events were reported in one of seven patients (14.3%) treated with agalsidase beta (75).

**Table 8:** WMLs reported between baseline and follow up in patients with FD treated with agalsidase beta.

Publication (Study)		Definition	Treatment (follow-up duration)	*N*	Patients with ≥1 event, *n* (%)	Outcome measure		*p* value**^a^**
Interventional studies								
Fellgiebel et al., 2014 (NCT00074984) (82)		NR	Agalsidase beta 1.0 mg/kg EOW (27 months)	25	NR	Mean change (SD): 0.7 (1.9)	Overall, there were no significant differences in WML burden between the agalsidase beta treated group and placebo groups at baseline or at follow-up. WML burden tended to increase in both the agalsidase beta treated group and the placebo group.	0.741
			Placebo (27 months)	16	NR	Mean change (SD): 1.0 (2.1)		
Ramaswami et al., 2019 (NCT00701415) (41)	Ramaswami et al., 2019 (NCT00701415) (41)	NR	Agalsidase beta 0.5 mg/kg EOW (5 years)	16	NR	No new WMLs. WMLs of unclear etiology observed in 1 patient remained stable.		NR
			Agalsidase beta 1.0 mg/kg q4w (5 years)	15				
	van der Veen et al., 2022 (75)	WML (all Fazekas 1)	Treated (10 years) 0.5 mg/kg biweekly Or 1.0 mg/kg once a month Switch to full dose (1 mg/kg biweekly)	7^b^	1 (14.3)	NR		NR
			Untreated (10 years)	22	6 (27.3)	One patient had a lacunar infarction		
Observational studies								
Rombach et al., 2012 (64)		Development of WML and stroke	Agalsidase beta 0.2 mg/kg EOW (≥12 months)	59	NR	The HR for developing WML and stroke was 0.74 (95% CI: 0.60, 0.90) per 10 nM lyso-GL3 decrease during treatment		0.009
			Agalsidase beta 1.0 mg/kg EOW (≥12 months)					
			Agalsidase alfa 0.2 mg/kg EOW (≥12 months)					
Observational switch studies								
Politei et al., 2016 (68)		NR	Agalsidase beta 1.0 mg/kg EOW (144 months)	12	1 (8.3)	The analysis of brain MRI in our study showed worsening of WML in four patients, three of whom (two males) had already presented with earlier cerebrovascular compromise, pre-existent to the initiation of ERT, while a male patient showed new lesions after 3 years of treatment with agalsidase alfa		NR
			Agalsidase alfa 0.2 mg/kg EOW (144 months)					

Data were extracted from publications with the most complete dataset.

EOW, every other week; ERT, enzyme replacement therapy; HR, hazard ratio; lyso-GL3, globotriaosylsphingosine; MRI, magnetic resonance imaging; NR, not reported; and WML, white matter lesion.

^a^ *p* values refer to comparison between follow-up and baseline values, unless otherwise stated.

^b^ Includes seven patients from Ramaswami et al., 2019 (41), with 10-year follow-up.

## 5 Discussion

The natural history of target organ injury in FD progresses slowly in patients with the classic phenotype and even more slowly in those with later-onset FD. Chronologically, the first natural history events in males with classic disease are typically kidney related (84). Structural tissue changes in the heart often occur as an early manifestation of FD due to accumulation of GL3 and related sphingolipids in cardiac cells and tissues, which are associated with progression to cardiac events (2). Subsequent cardiac events usually occur later in classic FD (median age 45 years in males and 54 years in females) and even later in later-onset disease (85). In assessing the natural disease progression, it takes approximately four decades for patients with kidney disease to reach end-stage kidney disease and even longer for cardiac events to occur (84, 85). Moreover, the early detection and management of cardiovascular risk factors is associated with low adverse outcomes in patients with FD (86). Agalsidase beta was first approved in the United States in 2003 and in Europe in 2001 based on the results from a phase 3, multi-center, randomized, placebo-controlled trial and its extension study in which 58 participants with classical FD received 1 mg/kg agalsidase beta EOW (21, 87–89). The primary outcome was the percentage of patients whose tissues were completely cleared of GL3 deposits at 20 weeks follow-up (89). The study reported complete or almost complete clearance of renal endothelial GL3 deposits in 69% of patients in the agalsidase beta group, compared with 0.0% of patients in the placebo group (89). Furthermore, evaluation of heart capillary endothelial cells showed that most agalsidase beta patients with biopsies at baseline and Month 6 had nearly complete clearance of GL3 from the capillary endothelium (89). With sample size limitations due to the low prevalence of FD, in addition to the timeline of progression to clinical events, a comprehensive evaluation of clinical studies is necessary to evaluate the effect of agalsidase beta on clinical outcomes. Since the approval and marketing authorization of agalsidase beta, dating back more than 20 years, observational studies have generated a body of evidence, complementary to that from clinical trials, that provides insights into the effectiveness on different target organ outcomes (kidney, heart, and brain). A meta-analysis published using an expansive evidence base has conclusively shown the clinical benefit of agalsidase beta on renal function (90). This SLR attempts to systematically present published data over the last two decades on the clinical efficacy and effectiveness of agalsidase beta on cardiovascular and cerebrovascular outcomes in patients with FD.

Overall, the SLR comprised 41 studies published since 2000, including four RCTs, five interventional studies, and 32 observational studies. The majority of studies investigated the regular dose of agalsidase beta, i.e., 1.0 mg/kg EOW, and included adult patients. Other study characteristics, such as numbers of patients, follow-up time, FD phenotype, and disease severity were more heterogeneous, providing a diverse evidence base. Published data identified in this SLR showed that agalsidase beta therapy may lead to stabilization or improvement in cardiac wall hypertrophy and mass, with data from 13 studies showing statistically significant reductions in IVST and/or LVPWT, or cardiac mass. While most studies which assessed cardiac mass utilized transthoracic echocardiography, a significant reduction in cardiac mass was also reported where gold-standard cardiac MRI was used (47). Data from several studies, including one with 10-year follow-up (40), also showed stabilization of cardiac wall thickness and mass with agalsidase beta treatment**.** These findings are consistent with a recent meta-analysis of MRI studies which found ERT was associated with stabilization of LVM and maximum left ventricular wall thickness (91).

The available evidence suggests a benefit of early initiation of ERT with agalsidase beta before the occurrence of clinical events. For example, an observational study of patients in the Fabry Registry demonstrated significant reduction in cardiac mass (LVM) in patients treated with agalsidase beta compared with untreated patients (48). However, this effect was only evident in the subgroup that initiated treatment between 18 and 29 years of age, while groups that initiated agalsidase beta when they were older showed no significant difference vs. controls (48). In the limited studies of agalsidase beta conducted exclusively in pediatric populations, improvement or stabilization of cardiac structure or mass was also evident up to 10 years after treatment initiation in childhood (41, 75). Data also showed that patients with less advanced disease may also derive more benefit from agalsidase beta. Three studies stratified cardiac structure and mass outcomes according to whether there was evidence of myocardial fibrosis on MRI (51, 66, 76). Those patients without fibrosis were more likely to have significant reductions in cardiac wall thickness or mass than those with detectable fibrosis, even of mild severity (51, 66, 76). A greater treatment benefit with agalsidase beta was also reported in patients without renal impairment at study baseline (52). This shows that patients with impaired renal function may display more severe cardiac hypertrophy, as assessed by LVPWT, and may develop cardiovascular progression despite being treated with agalsidase beta (52). Likewise, cardiac disease may also affect renal function, in a reciprocal relationship termed cardio-renal syndrome (92). These findings highlight the importance of early and routine monitoring of cardiac outcomes in patients with FD, especially when renal function is impaired. This is also consistent with a recent study that was published after the cut-off date for this SLR, which demonstrated a reduction in the incidence of cardiac complications and cardiac mortality with early ERT intervention in patients with FD (86).

Analysis of cardiac events, including MI, angina, and implantable cardioverter defibrillator/pacemaker implantation, indicated a generally low rate of these events (0.0%–27.3%) in patients treated with agalsidase beta. This suggests that agalsidase beta treatment may slow the progression to cardiac events in patients with FD. Incidence of new-onset arrhythmias or AF in interventional studies with up to 3 years of follow-up was generally low (3.9%–12.5%) and comparable to that reported for placebo (9.7%) (39, 44, 45). However, monitoring of arrhythmic events was likely based on electrocardiograms and Holter monitoring at scheduled study visits, rather than long-term monitoring using event monitors and implantable loop recorders, and therefore, these may be an underestimation of actual rates.

WMLs are frequently detected in brain scans of patients with FD (93); however, there was no evidence of improvement or worsening in WML burden in patients receiving agalsidase beta. It should be noted that the clinical consequence of WML in patients with FD is poorly understood and the interpretation of these data is therefore challenging. In addition, ERT does not cross the blood-brain barrier. Other central nervous system events assessed in this SLR included cerebrovascular ischemic events of stroke and TIA, which may have been influenced by supportive therapy. A low risk of these events was also demonstrated (0.0%–18.9% over follow-up periods of ≥12 months to 10 years), with similar or lower rates than comparators in interventional studies.

Strengths of this SLR include comprehensive searches of peer-reviewed literature and the involvement of two independent researchers in the study selection and data extraction, in accordance with PRISMA guidelines (30, 31). In addition, most studies included in the SLR were considered to be of good quality with a low risk of bias based on established criteria (32, 33). Previous SLRs of the efficacy of ERT with agalsidase beta in FD have focused on efficacy in specific study populations (e.g., males, females, pediatrics) (94, 95) and, to our knowledge, this is the first to focus on cardiac and cerebrovascular outcomes. A limitation, however, is that the majority of the studies identified were observational studies and many of these had retrospective designs, which may have influenced quality. Differences in the methodologies used in different studies to assess cardiac parameters (e.g., MRI vs*.* echocardiography), must also be considered when interpreting these results (96). Some of the studies did not clearly describe whether the study population had classic or later-onset FD, which may confound the measured outcome. In terms of study outcomes, specific cardiac outcomes, such as heart failure and syncope, were not extracted unless part of composite cardiac outcomes. Overall, the heterogeneity between studies in terms of populations, outcomes, follow-up, and imaging modalities limit the strength of the results. In addition, limited data were available from pediatric patients with FD; however, the lack of large controlled prospective trials in adults or pediatrics was not unexpected given the rarity of FD. Finally, the study selection criteria did not include conference materials, meaning that some recently completed studies may have been excluded, and the search was completed in 2022, excluding more recent publications.

Data from other existing and emerging therapies may be of interest in a future review to assess the relative impact of treatment options on patients with FD. However, comparative assessments prove challenging as a result of phenotypic heterogeneity and varying methods of measurement (e.g., echocardiography or cardiac MRI) (96). Future studies with systematic data collection in a controlled setting are required to corroborate and strengthen the evidence shown here. In addition, the search strategy utilized for this SLR was not designed to capture studies on the effects of agalsidase beta on GL3 and related sphingolipid deposits in cardiac tissue, or its effects on endothelial dysfunction and inflammation, although these data are limited. Data from an RCT of agalsidase beta in patients with classic FD showed significantly greater clearance of microvascular endothelial deposits of GL3 in cardiac tissue vs. placebo (89). However, preliminary data from a small cohort study of older patients with more severe cardiac disease at baseline did not find evidence of any improvement in coronary microvasculature dysfunction (based on myocardial blood flow and coronary flow reserve) with agalsidase beta treatment (54). Further studies on this in broader patient populations are warranted. Future studies on the effect of agalsidase beta on specific cardiac biomarkers such as high-sensitivity cardiac troponin I, an independent marker of major adverse cardiovascular events, would also be of value in patients with FD (97–99). Available data from a small cohort study of ERT (agalsidase alfa or agalsidase beta) have demonstrated that established ERT use does not normalize high-sensitivity troponin T levels in patients with advanced, but stable, disease (100). While a prospective observational clinical study of 36 ERT-naïve patients reported that in patients with high-sensitivity troponin I levels within the normal range at baseline (*n* = 20), levels remained within this range after agalsidase alfa treatment; whereas in patients with abnormally high levels at baseline (*n* = 16), levels generally remained stable (101). However, patients with FD who are eligible to receive ERT in clinical practice are likely to have more advanced disease.

Nonetheless, our study attempts to provide a comprehensive assessment of the available published evidence to evaluate the impact of agalsidase beta on clinical benefit in the cardiovascular and cerebrovascular systems of patients with FD. In conclusion, this SLR supports that agalsidase beta treatment may be associated with stabilization or regression of cardiac structural parameters and mass, and a reduction in cardiac and cerebrovascular events relative to controls. For patients who have already experienced cardiac disease progression, stabilization of cardiac structure and mass on agalsidase beta treatment may be a sufficient demonstration of treatment effect. Overall, agalsidase beta may be most effective if initiated when the progression is reversible prior to the onset of advanced cardiac fibrosis. How the impact of emerging therapies compares with that of agalsidase beta on cardiac and cerebrovascular aspects of FD remains a topic of interest in this field.

## Data Availability

The original contributions presented in the study are included in the article/Supplementary Material, further inquiries can be directed to the corresponding author.
