# Supplementary material for: A systematic literature review to evaluate the cardiac and cerebrovascular outcomes of patients with Fabry disease treated with agalsidase Beta
Source: Front Cardiovasc Med. 2025 Jan 21;11:1415547. doi: 10.3389/fcvm.2024.1415547 (PMC11790562; doi:10.3389/fcvm.2024.1415547)
Supplement: Supplementary file 1 [file Datasheet1.pdf]

Supplementary Material

A systematic literature review to evaluate the cardiac and cerebrovascular outcomes of patients with Fabry disease treated with agalsidase beta

Supplementary Table 1. Search strategy for Embase (January 2000 to March 2019).

Database: Embase 1974 to 2019 March 13<br>Search executed: March 13, 2019			
**No.**	**Criteria**	**Search terms**	**Hits**
1	Population terms	exp Fabry disease/	6,726
2		(fabry* or angiokeratoma or alpha galactosidase deficiency syndrome).mp.	8,602
3		1 or 2	8,602
4	Intervention terms	exp agalsidase beta/	1,024
5		(fabrazyme or agalsidase or (agalsidase adj3 beta)).mp.	1,409
6		4 or 5	1,409
7	Immunogenicity terms	exp immunogenicity/	56,472
8		exp neutralizing antibody/	32,842
9		exp drug antibody/	28,912
10		exp monoclonal antibody/	510,511
11		(immunogen* or anti drug antibod* or ADA* or neutraliz* or (serum-mediated adj3 inhibition) or immunoglobulin or IgG).mp.	1,610,399
12		or/7-11	2,005,919
13	Combined criteria	3 and (6 or 12)	1,540
14	Limits	limit 13 to (human and english language)	1,271
15	Date restriction	limit 14 to yr="2000 -Current"	1,255

Supplementary Table 2. Search strategy for MEDLINE (January 2000 to March 2019).

Database: Ovid MEDLINE(R) and Epub Ahead of Print, In-Process & Other Non-Indexed Citations, Daily and Versions(R) 1946 to March 13, 2019<br>Search executed: March 13, 2019			
**No.**	**Criteria**	**Search terms**	**Hits**
1	Population terms	exp Fabry disease/	3,239
2		(fabry* or angiokeratoma or alpha galactosidase deficiency syndrome).mp.	8,179
3		1 or 2	8,179
4	Intervention terms	(fabrazyme or agalsidase or (agalsidase adj3 beta)).mp.	436
5	Immunogenicity terms	exp Immunogenicity, Vaccine/	785
6		exp Antibodies, Neutralizing/	9,155
7		exp Antibodies, Monoclonal/	219,642
8		(immunogen* or anti drug antibod* or ADA* or neutraliz* or (serum-mediated adj3 inhibition) or immunoglobulin or IgG).mp.	1,177,334
9		or/5-8	1,343,265
10	Combined criteria	3 and (4 or 9)	548
11	Limits	limit 10 to (english language and humans)	373
12	Date restriction	limit 11 to yr="2000 -Current"	357

Supplementary Table 3. Search strategy for Cochrane Central Register of Controlled Trials (January 2000 to March 2019).

Database: EBM Reviews - Cochrane Central Register of Controlled Trials February 2019<br>Search executed: March 13, 2019			
**No.**	**Criteria**	**Search terms**	**Hits**
1	Population terms	exp Fabry disease/	60
2		(fabry* or angiokeratoma or alpha galactosidase deficiency syndrome).mp.	212
3		1 or 2	212
4	Intervention terms	(fabrazyme or agalsidase or (agalsidase adj3 beta)).mp.	59
5	Immunogenicity terms	exp Antibodies, Neutralizing/	310
6		exp Antibodies, Monoclonal/	7,007
7		(immunogen* or anti drug antibod* or ADA* or neutraliz* or (serum-mediated adj3 inhibition) or immunoglobulin or IgG).mp.	49,112
8		or/5-7	54,388
9	Combined criteria	3 and (4 or 8)	63
10	Limits	limit 9 to english language	53
11	Date restriction	limit 10 to yr="2000 -Current"	53

Supplementary Table 4. Search strategy for Embase (March 2019 to June 2022).

**Database: Embase. Accessed via ProQuest Dialog. March 2019 - June 2022<br>Search executed: June 15, 2022**			
**No.**	**Criteria**	**Search terms**	**Hits**
1	Population terms	emb("fabry disease")	8,954
2		((fabry* OR angiokeratoma OR "alpha galactosidase deficiency syndrome"))	14,847
3		S2 OR S1	14,847
4	Intervention terms	emb("agalsidase beta")	1,200
5		((fabrazyme or agalsidase or (agalsidase NEAR/3 beta)))	1,648
6		S5 OR S4	1,648
7	Immunogenicity terms	emb(immunogenicity)	77,861
8		emb("neutralizing antibody")	51,958
9		emb("drug antibody")	6,517
10		emb("monoclonal antibody")	251,161
11		((((immunogen* OR "anti drug antibod*" OR ADA* OR neutraliz* OR (serum-mediated NEAR/3 inhibition) OR immunoglobulin OR IgG))))	2,554,513
12		S11 OR S10 OR S9 OR S8 OR S7	2,736,710
13	Combined criteria	S3 AND (S6 OR S12)	2,207
14	Limits	limit S13 to (Human and English language)	1,806
15	Date restriction	limit S14 to yr=From March 13 2019-Current	381

Supplementary Table 5. Search strategy for MEDLINE (March 2019 to June 2022).

Database: PubMed MEDLINE. March 2019 - June 2022<br>Search executed: June 13, 2022			
**No.**	**Criteria**	**Search terms**	**Hits**
1	Population terms	"Fabry Disease"[Mesh]	3,939
2		fabry*[all] OR angiokeratoma[all] OR "alpha galactosidase deficiency syndrome"[all]	12,227
3		#1 OR #2	8,179
4	Intervention terms	("fabrazyme"[all] OR "agalsidase"[all] OR ("agalsidase"[all] AND "beta"[all]))	522
5	Immunogenicity terms	"Immunogenicity, Vaccine"[Mesh]	3,034
6		"Antibodies, Neutralizing"[Mesh]	15,923
7		"Antibodies, Monoclonal"[Mesh]	265,431
8		(immunogen* OR "anti drug antibod*" OR "ADA" OR neutraliz* OR ("serum-mediated" AND inhibition) OR immunoglobulin or IgG)	1,192,281
9		#5 OR #6 OR #7 OR #8	1,192,281
10	Combined criteria	#3 AND (#4 OR #9)	701
11	Limits	limit #10 to (Humans and English Language)	511
12	Date restriction	limit #11 to yr= From March 13 2019-Current	72

Supplementary Table 6. Search strategy for Cochrane Central Register of Controlled Trials (March 2019 to June 2022).

Database: Cochrane Central Register of Controlled Trials. 2019 - 2022<br>Search executed: June 15, 2022			
**No.**	**Criteria**	**Search terms**	**Hits**
1	Population terms	MeSH descriptor: [Fabry Disease] explode all trees	78
2		(fabry* OR angiokeratoma OR "alpha galactosidase deficiency syndrome")	325
3		#1 OR #2	325
4	Intervention terms	(fabrazyme OR agalsidase OR (agalsidase near/3 beta))	89
5	Immunogenicity terms	MeSH descriptor: [Antibodies, Neutralizing] explode all trees	560
6		MeSH descriptor: [Antibodies, Monoclonal] explode all trees	15,637
7		(immunogen* OR (anti drug antibod*) OR ADA* OR neutraliz* OR (serum-mediated near/3 inhibition) OR immunoglobulin OR IgG)	104,299
8		#5 OR #6 OR #7	116,469
9	Combined criteria	#3 AND (#4 OR #8)	102
10	Date restriction	limit #9 to yr=2019-Current	12
	Unable to limit by language in Cochrane		

Supplementary Table 7. Cochrane risk of bias assessment tool[1].

Domain	Support for judgment	Review authors' judgment
Selection bias		
Random sequence generation	Describe the method used to generate the allocation sequence in sufficient detail to allow an assessment of whether it should produce comparable groups.	Selection bias (biased allocation to interventions) due to inadequate generation of a randomized sequence.
Allocation concealment	Describe the method used to conceal the allocation sequence in sufficient detail to determine whether intervention allocations could have been foreseen in advance of, or during, enrolment.	Selection bias (biased allocation to interventions) due to inadequate concealment of allocations prior to assignment.
Performance bias		
Blinding of participants and personnel<br>Assessments should be made for each main outcome (or class of outcomes)	Describe all measures used, if any, to blind study participants and personnel from knowledge of which intervention a participant received. Provide any information relating to whether the intended blinding was effective.	Performance bias due to knowledge of the allocated interventions by participants and personnel during the study.
Detection bias		
Blinding of outcome assessment<br>Assessments should be made for each main outcome (or class of outcomes)	Describe all measures used, if any, to blind outcome assessors from knowledge of which intervention a participant received. Provide any information relating to whether the intended blinding was effective.	Detection bias due to knowledge of the allocated interventions by outcome assessors.
Attrition bias		
Incomplete outcome data<br>Assessments should be made for each main outcome (or class of outcomes)	Describe the completeness of outcome data for each main outcome, including attrition and exclusions from the analysis. State whether attrition and exclusions were reported, the numbers in each intervention group (compared with total randomized participants), reasons for attrition/exclusions where reported, and any re-inclusions in analyses performed by the review authors.	Attrition bias due to amount, nature or handling of incomplete outcome data.
Reporting bias		
Selective reporting	State how the possibility of selective outcome reporting was examined by the review authors, and what was found.	Reporting bias due to selective outcome reporting.
Other bias		
Other sources of bias	State any important concerns about bias not addressed in the other domains in the tool. If particular questions/entries were pre-specified in the review's protocol, responses should be provided for each question/entry.	Bias due to problems not covered elsewhere in the table.

Supplementary Table 8. Newcastle–Ottawa quality assessment scale – case-control studies[2].

Domain	Response
Selection	
1. Is the case definition adequate?	Yes, with independent validation*<br>Yes (e.g. record linkage or based on self-reports)<br>No description
2. Representativeness of the cases	Consecutive or obviously representative series of cases*<br>Potential for selection biases or not stated
3. Selection of controls	Community controls*<br>Hospital controls<br>No description
4. Definition of controls	No history of disease (endpoint)*<br>No description of source
Comparability	
1. Comparability of cases and controls on the basis of the design or analysis	Study controls for X (select the most important factor)*<br>Study controls for any additional factor (this criterion could be modified to indicate specific control for a second important factor)*
Exposure	
1. Ascertainment of exposure	Secure record (e.g. surgical records)*<br>Structured interview where blind to case/control status*<br>Interview not blinded to case/control status<br>Written self-report or medical record only<br>No description
2. Same method of ascertainment for cases and controls	Yes*<br>No
3. Non-response rate	Same rate for both groups*<br>Non-respondents described<br>Rate different and no designation

A study can be awarded a maximum of one star for each numbered item within the selection and exposure categories. A maximum of two stars can be given for comparability.

Supplementary Table 9. Newcastle–Ottawa quality assessment scale – cohort studies [2].

Domain	Response
Selection	
1. Representativeness of the exposed cohort	Truly representative of the average _______________ (describe) in the community*<br>Somewhat representative of the average ______________ in the community*<br>Selected group of users (e.g. nurses, volunteers)<br>No description of the derivation of the cohort
2. Selection of the non-exposed cohort	Drawn from the same community as the exposed cohort*<br>Drawn from a different source<br>No description of the derivation of the non-exposed cohort
3. Ascertainment of exposure	Secure record (e.g. surgical records)*<br>Structured interview*<br>Written self-report<br>No description
4. Demonstration that outcome of interest was not present at start of study	Yes*<br>No
Comparability	
1. Comparability of cohorts on the basis of the design or analysis	Study controls for _____________ (select the most important factor)*<br>Study controls for any additional factor (these criteria could be modified to indicate specific control for a second important factor)*
Outcomes	
1. Assessment of outcome	Independent blind assessment*<br>Record linkage*<br>Self-report<br>No description
2. Was follow-up long enough for outcomes to occur	Yes (select an adequate follow up period for outcome of interest)*<br>No
3. Adequacy of follow up of cohorts	Complete follow up - all subjects accounted for*<br>Subjects lost to follow up unlikely to introduce bias - small number lost - >____% (select an adequate %) follow up, or description provided of those lost)*<br>Follow up rate <____% (select an adequate %) and no description of those lost<br>No statement

A study can be awarded a maximum of one star for each numbered item within the selection and outcomes categories. A maximum of two stars can be given for comparability.

Supplementary Table 10. Summary of study characteristics.

Study name	Other publications	Study design	N	Region	Multicenter	Switch	Adult or pediatric	Phenotype[a]
	**Interventional studies**							
Fabry Disease Clinical Trial Study Group<br><br>Banikazemi et al, 2007 (NCT00074984) [3]	Fellgiebel et al, 2014 [4]; Benichou et al, 2009 [5]	RCT; Phase IV	82	USA, Canada, Czech Republic, Hungary, Poland, UK	Yes	No	Adult	Classic
International Collaborative Fabry Study Group<br><br>Germain et al, 2015 (NCT00074971; NCT00196742) [6]	Wilcox et al, 2004 [7]; Germain et al, 2007 [8]; Benichou et al, 2009 [5]	RCT and open-label extension study; Phase III	52	USA, France, Netherlands, UK	Yes	No	Adult and pediatric	Classic
Ramaswami et al, 2019 (NCT00701415) [9]	Van der Veen et al, 2020 [10][b]	RCT; open-label Phase IIIb	31	Argentina, Brazil, Canada, Czech Republic, Netherlands, Norway, Poland, UK, USA	Yes	No	Pediatric	Classic (except 1 patient with later-onset)
Kalliokoski et al, 2006 [11]		Single arm study	10	Finland	No	No	Adult	Unclear
Spinelli et al, 2004 [12]		Single arm study	9	Italy	No	No	Adult	Classic
Tahir et al, 2007 (NCT00343577) [13]		Non-randomized prospective single-arm study	11[c]	USA	No	No	Adult	Classic or unknown/not reported
Vedder et al, 2007 (ISRCTN45178534) [14]		RCT	34	Netherlands	No	No	Adult	Unclear
Wraith et al, 2008 (NCT00074958) [15]		Single arm study	16	France, Poland, UK, USA	Yes	No	Pediatric	Classic
Wuest et al, 2011 [16]		Single arm study	14	Germany	NR	No	Adult	Unclear
	**Observational studies: Fabry Disease Registry (NCT00196742)**							
Germain et al, 2013 [17]		Prospective cohort	163	International	Yes	No	Adult	Unclear[d]
Hopkin et al, 2016 [18]			1,411	International	Yes	No	Adult and pediatric	Classic or unknown
Wanner et al, 2020 [19]			42/86[e]	International	Yes	No	Adult	Classic or unknown
	**Other observational studies**							
Beer et al, 2006 [20]		Prospective cohort	17	Germany	NR	No	Adult	Severe (likely classic)
Breunig et al, 2006 [21]	Weidemann et al, 2003 [22]; Weidemann et al, 2009 [23]; Weidemann et al, 2013 [24]		40[f]	Germany	No	No	Adult	Unclear
Collin et al, 2011 [25]			46	France	No	No	Adult	Classic
Elliott et al, 2006 [26]			34[g]	UK	No	No	Adult	Classic

Study name	Other publications	Study design	N	Region	Multicenter	Switch	Adult or pediatric	Phenotype[a]
Imbriaco et al, 2009 [27]	Messalli et al, 2012 [28]		16[i]	Italy	NR	No	Adult	Unclear[h]
Koskenvuo et al, 2008 [29]			9	Finland	No	No	Adult	Classic and likely classic
Lenders et al, 2020 [30]			26	Germany	No	No	Adult	Classic (except for 5 patients)
Pisani et al, 2005 [31]			9	Italy	Yes	No	Adult	Classic
Politei et al, 2014 [32]			6	Argentina	NR	No	Adult and pediatric	Classic
Tsurumi et al, 2021 (NCT00233870) [33]			332	Japan	Yes	No	Adult and pediatric	Classic and later onset
Tsurumi et al, 2021 (AGAL02904) [33]			64	Japan	Yes	No	Adult and pediatric	Classic and later onset
Arends et al, 2018 [34]		Retrospective cohort	387	Netherlands, UK, Germany, Canada	Yes	No	Adult	Classic and later onset
Kim et al, 2016 [35]			19	Korea	NR	No	Adult and pediatric	Classic and unknown
Motwani et al, 2012 [36]			66	England	No	No	Adult	Unclear
Riccio et al, 2021 [37]			53	Italy	No	No	Adult	Classic and later onset
Rombach et al, 2012 [38]			59[j‖]	Netherlands	No	No	Adult	Classic
Tsuboi, 2007 [39]		Case series	11	Japan	No	No	Adult	Classic and later onset
Siamopoulos, 2004 [40]			2	Greece	No	No	Adult	Classic[k]
Koeppe et al, 2012 [41]		Case-control	25	Germany	NR	No	Adult	Unclear
Machann et al, 2011 [42]			23	Germany	No	No	Adult	Unclear
	**Observational switch studies: CFDI registry**							
Lenders et al, 2021 [43]		Prospective cohort	78	Germany	Yes	Yes	Adult	Classic (except for two patients)
	**Other observational switch studies**							
Limgala et al, 2019 [44]		Open-label, comparative study	27	USA	No	Yes	Adult and pediatric	Classic, (except three patients with later onset)
Politei et al, 2016 [45]		Prospective and retrospective Cohort	12	Argentina	NR	Yes	Adult	Classic
Lin et al, 2014 [46]			9	Taiwan	No	Yes	Adult and pediatric	Classic and later onset
Ripeau et al, 2017 [47]			33	Argentina, Venezuela	Yes	Yes	Adult and pediatric	Unclear
Vedder et al, 2008 [48]			52	Netherlands, Germany	Yes	Yes	Adult	Classic, later onset, and unclear[l]
Pisani et al, 2013 [49]			10	Italy	No	Yes	Adult	Classic
Tsuboi & Yamamoto, 2012 [50]	Tsuboi & Yamamoto, 2014 [51]		11	Japan	No	Yes	Adult	Classic and later onset

Study name	Other publications	Study design	N	Region	Multicenter	Switch	Adult or pediatric	Phenotype[a]
Weidemann et al, 2014 [52]	Lenders et al, 2016 [53]; Kramer et al, 2018 [54]		112[m]	Germany	Yes	Yes	Adult	Classic, later onset, and unclear

Data were extracted from publications with the most complete dataset.

[a]Unknown phenotype: mutation details were not reported in the publication as stated by the authors; unclear phenotype: mutation details were not mentioned by the authors and cannot be inferred based on limited information available. [b]Include seven patients from Ramaswami et al, 2019 [9], with 10-year follow-up. [c]Includes two patients from Banikazemi et al, 2007 [3]. [d]Plasma α-galactosidase activity (nmol/hr/mL) were available in 25 patients, and ranged from 0 to 18. [e]42 patients were included in the cardiac analysis and 86 in the estimated glomerular filtration rate analysis. [f]Source: Weidemann et al, 2013 [24]. [g]Study included 24 matched controls. [h]Source: Messalli et al, 2012 [28]. [i]Median baseline a-Gal A enzymatic activity in males was 0.25 nmol/h/mL, and values ranged from 0.2 to 4.2 nmol/h/mL (normal range 4.0 to 21.9); enzyme activity at baseline in the three females ranged from 2.1 to 4.2 nmol/h/mL. [j]Includes some patients from Vedder et al, 2007 [14]. [k]Both patients had T385P mutation, which is classic in phenotype; plasma a-Gal A activity were 0.8 and 0.7 nmol/hour/mL, respectively. [l]Male phenotypes: 12 classic, 2 later onset, 14 mutations unknown. [m]Source: Kramer et al, 2018 [54].

NR, not reported; and RCT, randomized controlled trial.

Supplementary Table 11. Cochrane risk of bias assessment of randomized controlled trials[1].

Study	Sequence generation	Allocation concealment	Blinding	Incomplete outcome data	Selective outcome reporting	Other sources of bias
Banikazemi et al, 2007 (NCT00074984) [3]	Low risk	Low risk	Low risk	Low risk	Low risk	Low risk
Germain et al, 2015 (NCT00074971; NCT00196742) [6]	Unclear	Unclear	Low risk	Low risk	Low risk	Low risk
Ramaswami et al, 2019 (NCT00701415) [9]	Low	Low	Low	Low	Low	Low
Vedder et al, 2007 (ISRCTN45178534) [14]	Low risk	Low risk	Low risk	Low risk	Low risk	Low risk

Supplementary Table 12. Newcastle–Ottawa Scale for assessing the quality of case-control studies[2].

Study	Selection				Comparability	Exposure		
	Is the case definition adequate?	Representative-ness of the cases	Selection of controls	Definition of controls	Comparability of cases and controls on basis of design or analysis	Ascertainment of exposure	Same method of ascertainment for cases and controls	Non-response rate
Machann et al, 2011 [42]	*	*			*	*	*	*
Koeppe et al, 2012 [41]	*	*	*	*	*	*	*	*

*A study can be awarded a maximum of one star for each numbered item within the selection and exposure categories. A maximum of two stars can be given for comparability.

Supplementary Table 13. The Newcastle–Ottawa Scale for assessing the quality of cohort studies and non-randomized studies[2].

Study	Selection				Comparability	Outcome		
	Represent-ativeness of exposed cohort	Selection of non-exposed (or comparator) cohort	Ascertain-ment of exposure	Demonstration that outcome of interest was not present at start of study	Comparability of cohorts on basis of the design or analysis	Assessment of outcome	Was follow-up long enough for outcomes to occur	Adequacy of follow-up of cohorts
Arends et al, 2018 [34]	*	*	*	*	*	*	*	
Politei et al, 2014 [32]		NA	*	*	NA	*	*	*
Pisani et al, 2013 [49]	*	NA	*	*	NA	*	*	*
Beer et al, 2006 [20]		NA	*	*	NA	*	*	*
Breunig et al, 2006 [21]	*	NA	*	*	NA	*	*	*
Collin et al, 2011 [25]	*	*	*	*	*	*	*	
Elliott et al, 2006 [26]	*	NA	*	*	NA	*	*	*
Imbriaco et al, 2009 [27]	*	NA	*	*	NA	*	*	
Kalliokoski et al, 2006 [11]		NA	*	*	NA	*	*	*
Kim et al, 2016 [35]	*	NA	*	*	NA	*	*	*
Koskenvuo et al, 2008 [29]		NA	*	*	NA	*	*	
Lin et al, 2014 [46]		NA	*	*	NA	*	*	*
Motwani et al, 2012 [36]		NA	*	*	NA	*	*	*
Hopkin et al, 2016 [18]	*	NA	*	*	NA	*	*	
Germain et al, 2013 [55]	*	*	*	*	*	*	*	
Pisani et al, 2005 [31]		NA	*	*	NA	*	*	*
Politei et al, 2016 [45]		NA		*	NA		*	*
Ripeau et al, 2017 [47]	*	*	*	*	*	*	*	*
Rombach et al, 2012 [38]	*	*	*	*	*	*	*	*
Spinelli et al, 2004 [12]		NA	*	*	NA	*	*	*
Tahir et al, 2007 [13]		NA	*	*	NA	*	*	*
Tsuboi & Yamamoto, 2012 [50]		NA	*	*	NA	*	*	*
Vedder et al, 2008 [48]		*	*	*	**	*	*	

Study	Selection				Comparability	Outcome		
	Represent-ativeness of exposed cohort	Selection of non-exposed (or comparator) cohort	Ascertain-ment of exposure	Demonstration that outcome of interest was not present at start of study	Comparability of cohorts on basis of the design or analysis	Assessment of outcome	Was follow-up long enough for outcomes to occur	Adequacy of follow-up of cohorts
Weidemann et al, 2014 [52]	*	*	*	*	*	*	*	*
Wraith et al, 2008 [15]	*	NA	*	*	NA	*	*	*
Wuest et al, 2011 [16]	*	NA	*	*	NA	*	*	*
Lenders et al, 2020 [30]	*	*	*		*	*	*	*
Lenders et al, 2021 [43]	*	*	*	*	*	*	** 7 years	
Wanner et al, 2020 [19]	*		*			*	* 10 years	
Limgala et al, 2019 [44]	*	Drawn from a different source	*	*	*		* 1 year	No mention
Riccio et al, 2021 [37]	*	*	*	*	*	*	*3.5 years	No mention
Tsurumi et al, 2021 [33]	*	No description of the derivation of the non-exposed cohort	*	*		*	*3.7 years	No mention

*A study can be awarded a maximum of one star for each numbered item within the selection and exposure categories. A maximum of two stars can be given for comparability.

NA, not applicable.

Supplementary Table 14. Interventricular septum thickness and left ventricular posterior wall thickness in patients with Fabry disease treated with agalsidase beta.

Study	Subgroup	Treatment (follow-up duration)	Imaging modality	N	Mean (SD) IVS thickness (mm)			Mean (SD) LVPW thickness (mm)		
					Baseline value	Follow-up value	p value[a]	Baseline value	Follow-up value	p value[a]
**Interventional studies**										
Germain et al, 2015 (NCT00074971; NCT00196742) [6]	—	Agalsidase beta 1.0 mg/kg EOW (120 months)	Echo	50[b]	10.7 (2.3)	11.5 (3.1)	NR	10.6 (2.3)	11.7 (3.0)	NR
Kalliokoski et al, 2006 [11]	—	Agalsidase beta 1.0 mg/kg EOW (12 months)	Echo	7	Systolic: 15.7 (0.9)	Systolic: 17.3 (1.7)	0.19	Systolic: 17.7 (1.9)	Systolic: 18.0 (1.8)	0.69
					Diastolic: 10.9 (1.6)	Diastolic: 12.1 (2.1)	0.25	Diastolic: 10.3 (1.1)	Diastolic: 10.0 (1.0)	0.63
Spinelli et al, 2004 [12]	—	Agalsidase beta 1.0 mg/kg EOW (12 months)	Echo	9	14 (3)[c]	13.3 (2.7)[c] *At 6 months of follow-up*	<0.025	13.3 (1.6)	13 (2.5) *At 6 months of follow-up*	>0.05
						12.4 (1.2)[c] *At 12 months of follow-up*			12.4 (1.5) *At 12 months of follow-up*	
Ramaswami et al, 2019 (NCT00701415) [9]	—	Agalsidase beta 0.5 mg/kg EOW (5 years)	Echo	16[d]	NR	NR	NR	8 (1) [n = 22]	8 (1) [n = 21]	0.2814 (Changes unremarkable)
	—	Agalsidase beta 1.0 mg/kg q4w (5 years)		15[d]	NR	NR	NR			
**Observational studies**										
Breunig et al, 2006 — Breunig et al, 2006 [21]	eGFR <90 at baseline	Agalsidase beta 1.0 mg/kg EOW (23 months)	Echo	16	NR			14.0 (2.1)	13.4 (2.3)	0.22
	eGFR >90 at baseline			9				11.7 (1.0)	10.7 (0.7)	0.017
Breunig et al, 2006 — Weidemann et al, 2009 [23]	No fibrosis	Agalsidase beta 1.0 mg/kg EOW (36 months)	Echo	12	13.5 (1.4)	12.0 (1.4)	<0.01	13.0 (1.2)	11.5 (1.6)	<0.01
	Mild fibrosis			11	14.4 (1.8)	13.4 (1.4)	0.20	14.4 (2.2)	12.9 (1.8)	0.21
	Severe fibrosis			9	14.9 (3.0)	14.1 (2.0)	0.01	14.7 (2.7)	12.8 (2.3)	0.17
Breunig et al, 2006 — Weidemann et al, 2013 [24]	—	v 1.0 mg/kg EOW (72 months)	Echo	40	13.5 (2.0)	11.9 (1.8)	<0.0001	13.2 (2.0)	11.4 (2.1)	<0.0001
Koeppe et al, 2012 [41]	LE positive	v 1.0 mg/kg EOW (12 months)	Cardiac MRI	11	13.2 (3.7)[e]	12.9 (3.6)[e]	<0.05[f]	NR	NR	NR
	LE negative			14	10.3 (1.5)[e]	9.8 (1.6)[e]		NR	NR	NR
Imbriaco et al, 2009 (Source: Messalli et al, 2012) [28]	—	Agalsidase beta 1.0 mg/kg EOW (mean: 48 months)	Cardiac MRI	16	NR	NR	NR	16 (3)	13 (3)	<0.001

Study	Subgroup	Treatment<br>(follow-up duration)	Imaging modality	N	Mean (SD) IVS thickness (mm)			Mean (SD) LVPW thickness (mm)		
					Baseline value	Follow-up value	p value[a]	Baseline value	Follow-up value	p value[a]
Pisani et al, 2005 [31]	—	Agalsidase beta 1.0 mg/kg EOW (24 months)	Echo	9	No difference was found in RWT between Fabry patients and healthy subjects. Compared with non-Fabry dialysis patients, Fabry patients showed a non-significant increase in RWT. Of note, mean (SD) RWT for Fabry patients at baseline was 0.57 (0.19).[g]					
Politei et al, 2014 [32]	—	Agalsidase beta 1.0 mg/kg EOW (120 months)	Echo	6[b]	Cardiac assessment prior to start of agalsidase beta treatment demonstrated only one patient with an increased thickness of the IVS and LVPW, whereby the thickness of the IVS and the LVPW after 10 years of treatment with agalsidase beta was normal.					
Lenders et al, 2020 [30]	Females/<br>Males	Agalsidase alfa 0.2 mg/kg EOW (12 months)	Echo	62	Females: 11.2 ± 2.4<br>Males: 16.2 ± 6.5	Females: 13.3 ± 4.4<br>Males: 14.5 ± 5.8	Females: 0.1248<br>Males: 0.2043	NR	NR	NR
		Agalsidase beta 1.0 mg/kg EOW (12 months)			Females: 13.9 ± 4.7<br>Males: IVSd:14.6 ± 5.0	Females: 14.0 ± 3.4<br>Males: 15.8 ± 4.8	Females: 0.8517<br>Males: 0.4600	NR	NR	NR
Wanner et al. 2020 [19]	--	Agalsidase beta 0.9–1.1 mg/kg EOW (10 years; 3.6 years treatment period)	Echo	38	Pre-treatment slope: 0.33, 95% CI: 0.12, 0.54	Post-treatment slope: 0.01 mm/year, 95% CI: 0.20, 0.21	Pre–post difference = 0.07	Pre-treatment slope: 0.28, 95% CI: 0.10, 0.46	Post-treatment slope: −0.13 mm/year, 95% CI: −0.30, 0.04	Pre–post difference = <0.01
**Observational switch studies**										
Lin et al, 2014 [46]	—	Agalsidase beta 1.0 mg/kg EOW (0.7–88.6 months)<br>Agalsidase alfa 0.2 mg/kg EOW (≥12 months)	Echo	9[b]	Among the six patients with available echocardiographic data at baseline and after ERT, all six had decreasing or stable IVS thickness.			Among the six patients with available echocardiographic data at baseline and after ERT, four had decreasing LPW thickness.		
Pisani et al, 2013 [49]	—	Agalsidase beta 1.0 mg/kg EOW (48 months)	Cardiac MRI	10	NR			16 (4)	13 (4)	<0.05 (agalsidase beta *vs.* baseline)
		Agalsidase alfa 0.2 mg/kg EOW (20 months)<br>*Following switch from a*galsidase beta		10				NR	13 (3)	>0.05 (agalsidase beta *vs.* agalsidase alfa)
Ripeau et al, 2017 [47]	—	Agalsidase alfa 0.2 mg/kg EOW (24 months)<br>*Following switch from* Agalsidase beta	Echo	33[b]	10.6 (0.5)<br>*At switch*	11.0 (0.8)<br>*At 12 months of follow-up*<br>10.8 (0.6)<br>*At 24 months of follow-up*	NS for repeated measures ANOVA	10.2 (0.5)<br>*At switch*	9.8 (0.5)<br>*At 12 months of follow-up*<br>9.9 (0.5)<br>*At 24 months of follow-up*	NS for repeated measures ANOVA
Tsuboi & Yamamoto, 2012 [50]<br>(Source: Tsuboi & Yamamoto, 2014) [51]	—	Agalsidase alfa 0.2 mg/kg EOW (36 months)<br>*Following switch from* Agalsidase beta	Echo	11	12.1	10.8	0.0426	12.3	11.5	0.00236

Study		Subgroup	Treatment (follow-up duration)		Imaging modality	N	Mean (SD) IVS thickness (mm)			Mean (SD) LVPW thickness (mm)		
							Baseline value	Follow-up value	p value[a]	Baseline value	Follow-up value	p value[a]
Weide-mann et al, 2014	Weidemann et al, 2014 [52]	—	Regular dose group	Agalsidase beta 1.0 mg/kg EOW (11 months)	Echo	38	12.9 (3.1)	13.1 (3.3)	0.08	12.5 (2.1)	12.8 (2.9)	0.27
			Dose-reduction group	Agalsidase beta 0.5 mg/kg EOW (12 months)		29	12.8 (3.6)	12.5 (3.2)	0.41	11.2 (2.8)	11.0 (3.0)	0.52
			Switch group	Agalsidase alfa 0.2 mg/kg EOW (12 months)		38	12.5 (3.2)	12.3 (2.5)	0.05	12.8 (2.4)	12.2 (2.9)	0.13
	Kramer et al, 2018 [54]	—	Regular dose group	Agalsidase beta 1.0 mg/kg EOW (≥12 months)	Echo	37	12.7 (3.0)	12.7 (3.3)[h]	NR	NR		
				Agalsidase beta 1.0 mg/kg EOW (≥12 months)				12.8 (3.8)[i]				
			Switch group	Agalsidase alfa 0.2 mg/kg EOW (≥12 months)		38	13.2 (3.2)	12.8 (3.1)[h]				
				Agalsidase alfa 0.2 mg/kg EOW (≥12 months)				13.4 (3.4)[i]				
			Re-switch group	Agalsidase alfa 0.2 mg/kg EOW (≥12 months)		37	13.1 (3.2)	12.8 (3.8)[h]				
				Agalsidase beta 1.0 mg/kg EOW (≥12 months)				13.5 (3.4)[i]				
Lenders et al, 2021 [43]		—	Agalsidase beta regular dose group (1.0 mg/kg (>12 months)		Echo	17	Diastole: 12.0 (3.0)	Diastole: 12.0 (2.6)	—	—		
			Switch group (beta [>12 months] to alfa [0.2 mg/kg; >24 months])		Echo	22	Diastole: 12.1 (3.0)	Diastole: 13.0 (3.6)	NR	NR		
			Re-switch group (beta [>12 months] to alfa [0.2 mg/kg >24 months] to beta)			39	Diastole: 13.6 (3.2)	Diastole: 14.1 (3.2)	NR	NR		

Data were extracted from publications with the most complete dataset. eGFR units are mL/min/1.73 m^2 unless otherwise specified. Weidemann et al, 2003 [56] reported data from a shorter follow-up duration for Breunig et al, 2006 [21] and therefore was not included in the table. Weidemann et al, 2014 [52] and Lenders et al, 2016 [53] reported data from shorter follow-up durations (one year and two years of follow-up, respectively) for the same study compared to Kramer et al, 2018 [54] (four years of follow-up), and therefore were not included in the table. Imbriaco et al, 2009 [27] reported data from shorter follow-up for the same study compared to Messalli et al, 2012 [28], and therefore was not included in the table.

[a]p values refer to comparison between follow-up and baseline values, unless otherwise stated. [b]Population was a mix of adult and pediatric patients. [c]Calculated from Figure 1 in Spinelli et al. 2004 [12]. [d]Pediatric population. [e]End diastolic LV septal wall thickness. [f]LE positive *vs*. LE negative; NS between baseline and follow-up. [g]RWT was calculated as: (IVS thickness + LVPW thickness) / (end-diastolic internal diameter of the left ventricle). [h]After being stable on therapy for ≥12 months. [i]After being stable on therapy for another period of ≥12 months.

ANOVA, analysis of variance; CI, confidence interval; echo, echocardiography; eGFR, estimated glomerular filtration rate; EOW, every other week; ERT, enzyme replacement therapy; IVS, interventricular septum; LE, late enhancement (indicator of myocardial fibrosis); LPW, left posterior wall; LV, left ventricle; LVPW, left ventricle posterior wall; MRI, magnetic resonance imaging; NR, not reported; NS, not significant; q4w, every four weeks; RWT, relative wall thickness; and SD, standard deviation.

Supplementary Table 15. Left ventricular mass and left ventricular mass index in patients with Fabry disease treated with agalsidase beta.

Study	Subgroup	Treatment (follow-up duration)	Imaging modality	N	Mean (SD) LV mass (g)			Mean (SD) LVMI (g/m^2)		
					Baseline value	Follow-up value	p value[a]	Baseline value	Follow-up value	p value[a]
**Interventional studies**										
Kalliokoski et al, 2006 [11]	—	Agalsidase beta 1.0 mg/kg EOW (12 months)	Echo	7	275 (28)	292 (35)	0.27	151 (12)	156 (14)	0.37
Spinelli et al, 2004 [12]	—	Agalsidase beta 1.0 mg/kg EOW (12 months)	Echo	9	Significant decrease in LV mass (p<0.001)			183 (44.5)	174.5 (41.7)[b] *At 6 months of follow-up*	NR
									169.5 (37.2)[b] *At 12 months of follow-up*	<0.01
Vedder et al, 2007 (ISRCTN45178534) [14]	—	Agalsidase beta 0.2 or 1.0 mg/kg EOW (24 months)	Echo	16	Median (SD): 313 (91)	296 (76) *At 12 months of follow-up*	NS[c]	NR		
						308 (90) *At 24 months of follow-up*				
		Agalsidase alfa 0.2 mg/kg EOW (24 months)		18	Median (SD): 279 (101)	244 (89) *At 12 months of follow-up*				
						294 (87) *At 24 months of follow-up*				
Wuest et al, 2011 [16]	—	Agalsidase beta 1.0 mg/kg EOW (13 months)	Cardiac MRI	14	193 (47)	178 (51)	<0.05	102 (26)	94 (27)	<0.05
Ramaswami et al, 2019 (NCT00701415) [9]	—	Agalsidase beta 0.5 mg/kg EOW	Echo	16[d]	NR	NR	NR	33.2 (5.1) g/m$^{2.7}$ [n = 23]	31.4 (7.2) g/m$^{2.7}$ [n = 24]	Changes unremarkable
		Agalsidase beta 1.0 mg/kg q4w		15[d]	NR	NR	NR			
**Observational studies: Fabry Disease Registry (NCT00196742)**										
Germain et al, 2013 [55]	—	Agalsidase beta 1.0 mg/kg EOW (58.8 months)	Echo	115	LVM progressively increased during the untreated period, whereas LVM decreased or remained stable for those who initiated agalsidase beta treatment.			NR		
		Natural history (52.8 months)		48						
	Age 18–29	Agalsidase beta 1.0 mg/kg EOW (58.8 months)		31	Mean LVM slope (SEM): −3.6 (1.62) g/year		<0.0001			

Study	Subgroup	Treatment (follow-up duration)	Imaging modality	N	Mean (SD) LV mass (g)			Mean (SD) LVMI (g/m^2)		
					Baseline value	Follow-up value	p value[a]	Baseline value	Follow-up value	p value[a]
		Natural history (52.8 months)	Echo	15	Mean LVM slope (SEM): 9.5 (2.36) g/year					
	Age 30–39	Agalsidase beta 1.0 mg/kg EOW (58.8 months)		44	Mean LVM slope (SEM): 2.8 (2.20) g/year		0.1760			
		Natural history (52.8 months)		17	Mean LVM slope (SEM): 8.4 (3.55) g/year					
	Age 40–49	Agalsidase beta 1.0 mg/kg EOW (58.8 months)		23	Mean LVM slope (SEM): 3.4 (2.87) g/year		0.1691			
		Natural history (52.8 months)		7	Mean LVM slope (SEM): 13.4 (6.63) g/year					
	Age ≥50	Agalsidase beta 1.0 mg/kg EOW (58.8 months)		17	Mean LVM slope (SEM): 7.7 (4.48) g/year		0.4843			
		Natural history (52.8 months)		9	Mean LVM slope (SEM): 0.4 (9.41)					
**Other observational studies**										
Arends et al, 2018 [34]	Patients included in longitudinal analysis	Agalsidase beta 1.0 mg/kg EOW (12 months)	Echo	278	NR			• $\beta_{slope\ Replagal-Fabrazyme}$ for change in LVMI in the first year: −2.26 g/m$^{2.7}$, 95% CI: −5.39, 0.87; p = 0.15<br>• Odds ratio of a decrease in LVMI after one year of treatment adjusted for LVMI at baseline (agalsidase beta *vs*. agalsidase alfa): 2.27, 95% CI: 1.11, 4.86; p = 0.03		
		Agalsidase alfa 0.2 mg/kg EOW (12 months)								
Beer et al, 2006 [20]	—	Agalsidase beta 1.0 mg/kg EOW (12 months)	Cardiac MRI	17	184 (49)	168 (53)	0.003	NR		
	LE positive			8	211 (58)	195 (64)	0.161			
	LE negative			9	160 (23)	145 (27)	0.008			
Breunig et al, 2006 – Weidemann et al, 2013 [24]	—	Agalsidase beta 1.0 mg/kg EOW (72 months)	Echo	40	270 (87)	224 (71)	<0.0001	NR		
Breunig et al, 2006 – Weidemann et al, 2009 [23]	No fibrosis	Agalsidase beta 1.0 mg/kg EOW (36 months)	Echo	12	238 (42)	202 (46)	<0.01			
	Mild fibrosis			11	275 (62)	244 (65)	0.31			
	Severe fibrosis			9	303 (84)	247 (45)	0.24			
Collin et al, 2011 [25]	Treated	Agalsidase beta 1.0 mg/kg EOW (54 months)	Cardiac MRI	30	NR			124 (66)	90 (37)	0.0021
								Adjusted slope (mean standard error) for change in LVMI per year: −7.8 (2.3) g/m^2/year; p<0.005		
Elliott et al, 2006 [26]	—	Agalsidase beta 1.0 mg/kg EOW (17.1 months)	Echo	5	NR			234 (81.3)	Change from baseline: 0.4 (37.8)	NS

Study	Subgroup	Treatment (follow-up duration)	Imaging modality	N	Mean (SD) LV mass (g)			Mean (SD) LVMI (g/m^2)		
					Baseline value	Follow-up value	p value[a]	Baseline value	Follow-up value	p value[a]
Kim et al, 2016 [35]	Males	Agalsidase beta 1.0 mg/kg EOW, with some reduction due to shortage (mean: 7.9 years)	Echo	11[e]	NR			59.7 (26.2) g/m$^{2.7}$	57 (22.4) g/m$^{2.7}$	NS
	Females	Agalsidase beta 1.0 mg/kg EOW, with some reduction due to shortage (mean: 9 years)		4[e]	One patient had LVH, which was stable while on agalsidase beta			NR		
Koeppe et al, 2012 [41]	LE positive	Agalsidase beta 1.0 mg/kg EOW (12 months)	Cardiac MRI	11	NR			108.7 (28.7)	102.7 (28.1)	<0.05[f]
	LE negative			14				84.6 (11.9)	79.9 (16.2)	
Koskenvuo et al, 2008 [29]	—	Agalsidase beta 1.0 mg/kg EOW (24 months)	Cardiac MRI	8	Difference between means at 1 year *vs*. baseline: −7.5, 95% CI: −27.4, 12.5		0.75	NR		
					Difference between means at 2 years *vs*. baseline: −2.9, 95% CI: −21.5, 16.7		0.96			
Machann et al, 2011 [42]	—	Agalsidase beta 1.0 mg/kg EOW (14 months)	Cardiac MRI	8	215 (55)	185 (45)	0.012	NR		
Imbriaco et al, 2009 [27] (Source: Messalli et al, 2012) [28]	—	Agalsidase beta 1.0 mg/kg EOW (mean: 48 months)	Cardiac MRI	16	187 (59)	149 (44)	<0.001	NR		
Motwani et al, 2012 [36]	—	Agalsidase beta 1.0 mg/kg EOW (mean: 3 years)	Echo	66	NR			116 (28)	113 (26)	<0.001
	LVH at baseline			42				135 (13)	133 (13)	<0.01
	No LVH at baseline			24				82 (6)	82 (5)	0.79
	Males			44				123 (2)	120 (26)	<0.001
	Females			22				101 (22)	98 (20)	<0.001
Pisani et al, 2005 [31]	—	Agalsidase beta 1.0 mg/kg EOW (24 months)	Echo	9	NR			73 (29)	Fabry patients showed a greater LVMI than healthy subjects (measured in g/m$^{2.7}$). Compared with non-Fabry dialysis patients, Fabry patients showed a non-significant increase in LVMI. After 24 months of agalsidase beta treatment, LVMI did not change compared with basal	

Study	Subgroup	Treatment (follow-up duration)	Imaging modality	N	Mean (SD) LV mass (g)			Mean (SD) LVMI (g/m^2)		
					Baseline value	Follow-up value	p value[a]	Baseline value	Follow-up value	p value[a]
									values. During the 24 months of therapy, progression of LVMI decreased in all except two patients. Average percentage of increase per year in LVMI was 6% during the 24 months preceding agalsidase beta initiation and 3% during the 3 years of treatment. This also was shown by comparison (p = 0.06) of LVMI curve slopes for the pretreatment period (0.98 ± 0.017) with those obtained during the treatment period (0.46 ± 0.960).	
Politei et al, 2014 [32]	—	Agalsidase beta 1.0 mg/kg EOW (120 months)	Echo	6[e]	There were no cases of progression to LVH after the study, whereby the only patient with abnormal values returned to the normal range for these parameters.			The LVMI was normal in four male patients and pathological in the female patients on commencement of the study. No progression to abnormal values was registered in the male patients after 10 years on agalsidase beta. One of the heterozygous patients showed no change and the other a clear reduction in LVMI, but without registering normal values.		
**Observational switch studies**										
Lin et al, 2014 [46]	—	Agalsidase beta 1.0 mg/kg EOW (0.7–88.6 months)<br>Agalsidase alfa 0.2 mg/kg EOW (≥12 months)	Echo	9[e]	NR			Among the six patients with available echocardiographic data at baseline and after ERT, all six experienced reductions of the LVMI (measured in g/m$^{2.7}$)		
Pisani et al, 2013 [49]	—	Agalsidase beta 1.0 mg/kg EOW (48 months)	Cardiac MRI	10	106 (32)	73 (24)	<0.05	NR		
		Agalsidase alfa 0.2 mg/kg EOW (20 months)<br>*Following switch from* agalsidase beta		10	NR	70 (24)	>0.05			
Ripeau et al, 2017 [47]	—	Agalsidase alfa 0.2 mg/kg EOW (24 months)	Echo	33[e]	NR			106.3 (6.2)<br>*At switch*	105.9 (7.7)<br>*At 12 months of follow-up*	NS

Study	Subgroup	Treatment (follow-up duration)	Imaging modality	N	Mean (SD) LV mass (g)			Mean (SD) LVMI (g/m^2)		
					Baseline value	Follow-up value	p value[a]	Baseline value	Follow-up value	p value[a]
		Following switch from Agalsidase beta							106.9 (7.0) *At 24 months of follow-up*	
Tsuboi & Yamamoto, 2012 [50] (Source: Tsuboi & Yamamoto, 2014) [51]	—	Agalsidase alfa 0.2 mg/kg EOW (36 months) *Following switch from* agalsidase beta	Echo	11	NR			58.1 g/m$^{2.7}$	50.7 g/m$^{2.7}$	0.0451
Vedder et al, 2008 [48]	Antibody positive	Agalsidase beta 1.0 mg/kg EOW (12 months)	Echo	6	Difference between medians (range) between baseline and follow-up: −77 (−175 to 12); p = 0.028		0.95[g]	NR		
	Antibody negative			8	Difference between medians (range) between baseline and follow-up: −65 (−234 to 19); p = 0.036					
Weidemann et al, 2014 [52]	—	Regular dose group: Agalsidase beta 1.0 mg/kg EOW (11 months)	Echo	38	NR			95.7 (35.8)	86.3 (24.9)	0.94
		Dose-reduction group: Agalsidase beta 0.5 mg/kg EOW (12 months)		29				91.5 (34.7)	87.1 (21.8)	0.69
		Switch group: Agalsidase alfa 0.2 mg/kg EOW (12 months)		38				82.8 (26.9)	80.3 (14.7)	0.26

Study	Subgroup	Treatment (follow-up duration)	Imaging modality	N	Mean (SD) LV mass (g)			Mean (SD) LVMI (g/m^2)		
					Baseline value	Follow-up value	p value[a]	Baseline value	Follow-up value	p value[a]
Lenders et al, 2020 [30]	Naïve/long-term	Agalsidase alfa 0.2 mg/kg EOW or (12 months)	Echo	62	At recruitment, two patients who were ERT-naïve at baseline had LVH (50.0%)<br>At recruitment, 18 patients had LVH at baseline (56.3%), following long-term ERT treatment			NR	NR	NR
		Agalsidase beta 1.0 mg/kg EOW (12 months)			At recruitment, six patients who were ERT treatment naïve at baseline had LVH (54.5%)<br>At recruitment, eight patients had LVH at baseline (53.3%), following long-term ERT treatment			NR	NR	NR
Lenders et al, 2021 [43]	—	Regular dose group (1.0 mg/kg (>12 months) (88±25 months)	Echo	17	LVH: n = 7	n = 8	RR=1.17	NR	NR	NR
		Switch group (beta to alfa [0.2 mg/kg]) (88±25 months)		22	LVH: n = 7	N = 11	RR=1.29	NR	NR	NR
		Re-switch group (beta to alfa for 12 months) (88±25 months)		39	LVH: n = 24	N = 27	RR=1.48	NR	NR	NR
van der Veen et al, 2022 [10]	Treated	0.5 mg/kg biweekly or 1.0 mg/kg once a month<br><br>Switch to full dose (1 mg/kg biweekly)<br><br>(10 years)	Echo and cardiac MRI	7[d]	Echo measurements<br>80 (67–84) g/m^2<br>MRI measurements<br>53 (46–59) g/m^2		Both measurements<br>p = 0.02	NR	NR	NR
	Untreated	(10 years)		23[d]	Echo measurements<br>94 (59–149) g/m^2<br>MRI measurements<br>68 (53–99) g/m^2			NR	NR	NR

Data were extracted from publications with the most complete dataset. Weidemann et al, 2003 [56] reported data from a shorter follow-up duration for Breunig et al, 2006 [21] and therefore was not included in the table. Weidemann et al, 2014 [52] and Lenders et al, 2016 [53] reported data from shorter follow-up durations (1 year and 2 years of follow-up, respectively) for the same study compared to Kramer et al, 2018 [54] (4 years of follow-up), and therefore were not included in the table. Imbriaco et al, 2009 [27] reported data from shorter follow-up for the same study compared to Messalli et al, 2012 [28], and therefore was not included in the table. LVMI units are g/m^2 unless otherwise stated.

[a]p values refer to comparison between follow-up and baseline values, unless otherwise stated. [b]Calculated from Figure 1 in Spinelli et al, 2004 [12]. [c]After 12 and 24 months of treatment no reduction in LVM was seen, which was not different between the two treatment groups. [d]Pediatric population. [e]Population mixed adult and pediatric patients. [f]LE positive *vs.* LE negative. [g]Antibody positive *vs.* antibody negative.

Echo, echocardiography; EOW, every other week; ERT, enzyme replacement therapy; LE, late enhancement (indicator of myocardial fibrosis); LV, left ventricle; LVH, left ventricular hypertrophy; LVMI, left ventricle mass index; MRI, magnetic resonance imaging; NR, not reported; NS, not significant; SD, standard deviation; and SEM, standard error of the mean.

Supplementary Table 16. Other cardiac events reported between baseline and follow-up in adult patients with Fabry disease treated with agalsidase beta.

Study		Treatment (follow-up duration)		N	Angina events	Myocardial infarction events		Pacemaker/defibrillator implantation events		
					Patients with ≥1 event, n (%)	Definition	Patients with ≥1 event, n (%)	Definition	Patients with ≥1 event, n (%)	Comparative measures
**Interventional studies**										
Banikazemi et al, 2007 (NCT00074984) [3]		Agalsidase beta 1.0 mg/kg EOW (35 months)		51	0 (0.0)	NR	1 (2.0)	NR	NR	NR
		Placebo (35 months)		31	1 (3.2)		0 (0.0)			
Germain et al, 2015 (NCT00074971; NCT00196742) [6]		Agalsidase beta 1.0 mg/kg EOW (120 months)		52[a]	NR	NR	1 (1.9)	NR	NR	NR
Kalliokoski et al, 2006 [11]		Agalsidase beta 1.0 mg/kg EOW (12 months)		10	0 (0.0)	NR	NR	NR	NR	NR
Tahir et al, 2007 (NCT00343577) [13]		Agalsidase beta 1.0 mg/kg EOW (≥25 months)		11	NR	NR	NR	Intracardiac defibrillator devices	3 (27.3)[b]	NR
**Observational studies**										
Breunig et al, 2006	Breunig et al, 2006 [21]	Agalsidase beta 1.0 mg/kg EOW (23 months)		25	NR	Myocardial infarction with subsequent coronary intervention	1 (4.0)	Cardiac pacemaker implantation	2 (8.0)	NR
	Weidemann et al, 2013 [24]	Agalsidase beta 1.0 mg/kg EOW (72 months)		40	NR	NR	NR	ICD	1 (2.5)[c]	NR
		Natural history (45 years)		40					NR	
Tsuboi, 2007 [39]		Agalsidase beta (dose NR; follow-up time NR)		11	NR	NR	NR	NR	One patient receiving agalsidase beta underwent pacemaker implantation to treat high-grade bradycardia. Two patients received a pacemaker before starting agalsidase beta. A fourth patient received a pacemaker but no details are reported. No complications as a result of ERT were seen in the four patients who underwent pacemaker implantation.	
**Observational switch studies**										
Weidemann et al, 2014 (Source: Kramer et al, 2018) [54]		Regular dose group[d]	Agalsidase beta 1.0 mg/kg EOW (≥12 months)	37	NR	NR	NR	Pacemaker/ICD	5 (13.5)[e]	RR: 1.58, 95% CI: 0.99, 2.48; NS[f]
			Agalsidase beta 1.0 mg/kg EOW (≥12 months)							

Study	Treatment (follow-up duration)		N	Angina events	Myocardial infarction events		Pacemaker/defibrillator implantation events		
				Patients with ≥1 event, n (%)	Definition	Patients with ≥1 event, n (%)	Definition	Patients with ≥1 event, n (%)	Comparative measures
	Switch group[d]	Agalsidase alfa 0.2 mg/kg EOW (≥12 months)<br>Agalsidase alfa 0.2 mg/kg EOW (≥12 months)	38					7 (18.4)[e]	RR: 1.98, 95% CI: 1.39, 2.81; p<0.05[f]
	Re-switch group[d]	Agalsidase alfa 0.2 mg/kg EOW (≥12 months)<br>Agalsidase beta 1.0 mg/kg EOW (≥12 months)	37					6 (16.2)[e]	RR: 1.77, 95% CI: 1.17, 2.66; p<0.05[f]
Lenders et al, 2021 [43]	Regular dose group	Regular dose group (1.0 mg/kg (>12 months) (88±25 months)	17	NR	NR	NR	Pacemaker/ICD	**Baseline:** 0 (0.0)<br>**Follow-up:** 4 (21.1)	RR: N/A;<br>p = 0.1026
	Switch group	Switch group (beta to alfa [0.2 mg/kg]) (88±25 months)	22	NR	NR	NR	Pacemaker/ICD	**Baseline:** 2 (9.5)<br>**Follow-up:** 5 (26.3)	RR: 1.50, 95% CI: 0.58, 3.86
	Re-switch group	Re-switch group (beta to alfa for 12 months) (88±25 months)	39	NR	NR	NR	Pacemaker/ICD	**Baseline:** 2 (5.1)<br>**Follow-up:** 10 (25.6)	RR: 1.27, 95% CI: 0.69, 2.36

Data were extracted from publications with the most complete dataset. Benichou et al, 2009 [5] reports the pooled outcomes of Banikazemi et al, 2007 [3] and Germain et al, 2007 [8] clinical trials, and was therefore not included in this table. Weidemann et al, 2014 [52] and Lenders et al, 2016 [53] reported data from shorter follow-up durations (one year and two years of follow-up, respectively) for the same study compared to Kramer et al, 2018 [54] (four years of follow-up), and therefore were not included in the table.

[a]Population was a mix of adult and pediatric patients; [b]Three patients developed ventricular ectopy and received intracardiac defibrillator devices at 0 months, 12 months, and 14 months after starting ERT; [c]One patient had received an ICD 2 years prior to death, with several documented episodes of ventricular tachycardia adequately abolished by the device; [d]All patients had received one year of agalsidase beta 1.0 mg/kg EOW at baseline; [e]Calculated as the difference in the number of cumulative events between baseline and second long-term follow-up; [f]Increasing frequencies for implanted pacemakers between baseline examination and second follow-up (24 months) were observed across treatment groups, while the resulting relative risks were only significant in the switch and re-switch groups.

CI, confidence interval; EOW, every other week; ERT, enzyme replacement therapy; ICD, implantable cardioverter defibrillator; NR, not reported; NS, not significant; and RR, relative risk.

References

1. Higgins JP, Altman DG, Gøtzsche PC, Jüni P, Moher D, Oxman AD, et al. The Cochrane Collaboration's tool for assessing risk of bias in randomised trials. BMJ (Clinical research ed). 2011;343:d5928.
2. Wells GS, O'Connell D, Peterson J, Welch V, Losos M. The Newcastle-Ottawa Scale (NOS) for assessing the quality of nonrandomised studies in meta-analyses 2013 [November 04, 2022]. Available from: http://www.ohri.ca/programs/clinical_epidemiology/oxford.asp.
3. Banikazemi MB, J.;Waldek, S.;Wilcox, W. R.;Whitley, C. B.;McDonald, M.;Finkel, R.;Packman, S.;Bichet, D. G.;Warnock, D. G.;Desnick, R. J.;Fabry Disease Clinical Trial Study, Group. Agalsidase-beta therapy for advanced Fabry disease: a randomized trial. Ann Intern Med. 2007;146(2):77-86.
4. Fellgiebel A, Gartenschlager M, Wildberger K, Scheurich A, Desnick RJ, Sims K. Enzyme replacement therapy stabilized white matter lesion progression in Fabry disease. Cerebrovascular diseases (Basel, Switzerland). 2014;38(6):448-56.
5. Bénichou B, Goyal S, Sung C, Norfleet AM, O'Brien F. A retrospective analysis of the potential impact of IgG antibodies to agalsidase beta on efficacy during enzyme replacement therapy for Fabry disease. Molecular genetics and metabolism. 2009;96(1):4-12.
6. Germain DP, Charrow J, Desnick RJ, Guffon N, Kempf J, Lachmann RH, et al. Ten-year outcome of enzyme replacement therapy with agalsidase beta in patients with Fabry disease. Journal of medical genetics. 2015;52(5):353-8.
7. Wilcox WRB, M.;Guffon, N.;Waldek, S.;Lee, P.;Linthorst, G. E.;Desnick, R. J.;Germain, D. P.;International Fabry Disease Study, Group. Long-term safety and efficacy of enzyme replacement therapy for Fabry disease. Am J Hum Genet. 2004;75(1):65-74.
8. Germain DPW, S.;Banikazemi, M.;Bushinsky, D. A.;Charrow, J.;Desnick, R. J.;Lee, P.;Loew, T.;Vedder, A. C.;Abichandani, R.;Wilcox, W. R.;Guffon, N. Sustained, long-term renal stabilization after 54 months of agalsidase beta therapy in patients with Fabry disease. Journal of the American Society of Nephrology : JASN. 2007;18(5):1547-57.
9. Ramaswami U, Bichet DG, Clarke LA, Dostalova G, Fainboim A, Fellgiebel A, et al. Low-dose agalsidase beta treatment in male pediatric patients with Fabry disease: A 5-year randomized controlled trial. Molecular genetics and metabolism. 2019;127(1):86-94.
10. van der Veen SJ, Körver S, Hirsch A, Hollak CEM, Wijburg FA, Brands MM, et al. Early start of enzyme replacement therapy in pediatric male patients with classical Fabry disease is associated with attenuated disease progression. Molecular genetics and metabolism. 2022;135(2):163-9.
11. Kalliokoski RJ, Kantola I, Kalliokoski KK, Engblom E, Sundell J, Hannukainen JC, et al. The effect of 12-month enzyme replacement therapy on myocardial perfusion in patients with Fabry disease. Journal of inherited metabolic disease. 2006;29(1):112-8.
12. Spinelli L, Pisani A, Sabbatini M, Petretta M, Andreucci MV, Procaccini D, et al. Enzyme replacement therapy with agalsidase beta improves cardiac involvement in Fabry's disease. Clinical genetics. 2004;66(2):158-65.
13. Tahir H, Jackson LL, Warnock DG. Antiproteinuric therapy and fabry nephropathy: sustained reduction of proteinuria in patients receiving enzyme replacement therapy with agalsidase-beta. Journal of the American Society of Nephrology : JASN. 2007;18(9):2609-17.
14. Vedder AC, Linthorst GE, Houge G, Groener JE, Ormel EE, Bouma BJ, et al. Treatment of Fabry disease: outcome of a comparative trial with agalsidase alfa or beta at a dose of 0.2 mg/kg. PLoS ONE [Electronic Resource]. 2007;2(7):e598.
15. Wraith JE, Tylki-Szymanska A, Guffon N, Lien YH, Tsimaratos M, Vellodi A, et al. Safety and efficacy of enzyme replacement therapy with agalsidase beta: an international, open-label study in pediatric patients with Fabry disease. The Journal of pediatrics. 2008;152(4):563-70, 70.e1.
16. Wuest WM, W.;Breunig, F.;Weidemann, F.;Koestler, H.;Hahn, D.;Wanner, C.;Beer, M. Right ventricular involvement in patients with Fabry's disease and the effect of enzyme replacement therapy. ROFO Fortschr Geb Rontgenstr Nuklearmed. 2011;183(11):1037-42.
17. Germain DP, Weidemann F, Abiose A, Patel MR, Cizmarik M, Cole JA, et al. Analysis of left ventricular mass in untreated men and in men treated with agalsidase-beta: data from the Fabry Registry. Genet Med. 2013;15(12):958-65.
18. Hopkin RJC, G.;Charrow, J.;Lemay, R.;Martins, A. M.;Mauer, M.;Ortiz, A.;Patel, M. R.;Sims, K.;Waldek, S.;Warnock, D. G.;Wilcox, W. R. Risk factors for severe clinical events in male and female patients with Fabry disease

treated with agalsidase beta enzyme replacement therapy: Data from the Fabry Registry. Molecular genetics and metabolism. 2016;119(1-2):151-9.
19. Wanner C, Feldt-Rasmussen U, Jovanovic A, Linhart A, Yang M, Ponce E, et al. Cardiomyopathy and kidney function in agalsidase beta-treated female Fabry patients: a pre-treatment vs. post-treatment analysis. ESC heart failure. 2020;7(3):825-34.
20. Beer M, Weidemann F, Breunig F, Knoll A, Koeppe S, Machann W, et al. Impact of enzyme replacement therapy on cardiac morphology and function and late enhancement in Fabry's cardiomyopathy. The American journal of cardiology. 2006;97(10):1515-8.
21. Breunig FW, F.;Strotmann, J.;Knoll, A.;Wanner, C. Clinical benefit of enzyme replacement therapy in Fabry disease. Kidney Int. 2006;69(7):1216-21.
22. Weidemann FB, F.;Beer, M.;Sandstede, J.;Turschner, O.;Voelker, W.;Ertl, G.;Knoll, A.;Wanner, C.;Strotmann, J. M. Improvement of cardiac function during enzyme replacement therapy in patients with Fabry disease: a prospective strain rate imaging study. Circulation. 2003;108(11):1299-301.
23. Weidemann FN, M.;Breunig, F.;Herrmann, S.;Beer, M.;Stork, S.;Voelker, W.;Ertl, G.;Wanner, C.;Strotmann, J. Long-term effects of enzyme replacement therapy on fabry cardiomyopathy: evidence for a better outcome with early treatment. Circulation. 2009;119(4):524-9.
24. Weidemann FN, M.;Stork, S.;Breunig, F.;Beer, M.;Sommer, C.;Herrmann, S.;Ertl, G.;Wanner, C. Long-term outcome of enzyme-replacement therapy in advanced Fabry disease: evidence for disease progression towards serious complications. J Intern Med. 2013;274(4):331-41.
25. Collin C, Briet M, Tran TC, Beaussier H, Benistan K, Bensalah M, et al. Long-term changes in arterial structure and function and left ventricular geometry after enzyme replacement therapy in patients affected with Fabry disease. European journal of preventive cardiology. 2011;19(1):43-54.
26. Elliott PM, Kindler H, Shah JS, Sachdev B, Rimoldi OE, Thaman R, et al. Coronary microvascular dysfunction in male patients with Anderson-Fabry disease and the effect of treatment with alpha galactosidase A. Heart. 2006;92(3):357-60.
27. Imbriaco M, Pisani A, Spinelli L, Cuocolo A, Messalli G, Capuano E, et al. Effects of enzyme-replacement therapy in patients with Anderson-Fabry disease: a prospective long-term cardiac magnetic resonance imaging study. Heart. 2009;95(13):1103-7.
28. Messalli G, Imbriaco M, Avitabile G, Russo R, Iodice D, Spinelli L, et al. Role of cardiac MRI in evaluating patients with Anderson-Fabry disease: assessing cardiac effects of long-term enzyme replacement therapy. La Radiologia medica. 2012;117(1):19-28.
29. Koskenvuo JW, Hartiala JJ, Nuutila P, Kalliokoski R, Viikari JS, Engblom E, et al. Twenty-four-month alpha-galactosidase A replacement therapy in Fabry disease has only minimal effects on symptoms and cardiovascular parameters. Journal of inherited metabolic disease. 2008;31(3):432-41.
30. Lenders M, Brand E. FAbry STabilization indEX (FASTEX): Clinical evaluation of disease progression in Fabry patients. Molecular genetics and metabolism. 2020;129(2):142-9.
31. Pisani A, Spinelli L, Sabbatini M, Andreucci MV, Procaccini D, Abbaterusso C, et al. Enzyme replacement therapy in Fabry disease patients undergoing dialysis: effects on quality of life and organ involvement. American journal of kidney diseases : the official journal of the National Kidney Foundation. 2005;46(1):120-7.
32. Politei J, Hernan A, Beatriz SA, Gustavo C, Antonio M, Eduardo T, et al. Fabry disease: multidisciplinary evaluation after 10 years of treatment with agalsidase Beta. JIMD reports. 2014;16:7-14.
33. Tsurumi M, Suzuki S, Hokugo J, Ueda K. Long-term safety and efficacy of agalsidase beta in Japanese patients with Fabry disease: aggregate data from two post-authorization safety studies. Expert opinion on drug safety. 2021;20(5):589-601.
34. Arends M, Biegstraaten M, Wanner C, Sirrs S, Mehta A, Elliott PM, et al. Agalsidase alfa versus agalsidase beta for the treatment of Fabry disease: an international cohort study. Journal of medical genetics. 2018;55(5):351-8.
35. Kim JHL, B. H.;Hyang Cho, J.;Kang, E.;Choi, J. H.;Kim, G. H.;Yoo, H. W. Long-term enzyme replacement therapy for Fabry disease: efficacy and unmet needs in cardiac and renal outcomes. J Hum Genet. 2016;61(11):923-9.
36. Motwani M, Banypersad S, Woolfson P, Waldek S. Enzyme replacement therapy improves cardiac features and severity of Fabry disease. Molecular genetics and metabolism. 2012;107(1-2):197-202.
37. Riccio E, Zanfardino M, Franzese M, Capuano I, Buonanno P, Ferreri L, et al. Stepwise shortening of agalsidase beta infusion duration in Fabry disease: Clinical experience with infusion rate escalation protocol. Molecular genetics & genomic medicine. 2021;9(5):e1659.

38. Rombach SM, Aerts JM, Poorthuis BJ, Groener JE, Donker-Koopman W, Hendriks E, et al. Long-term effect of antibodies against infused alpha-galactosidase A in Fabry disease on plasma and urinary (lyso)Gb3 reduction and treatment outcome. PLoS ONE [Electronic Resource]. 2012;7(10):e47805.
39. Tsuboi K. Enzyme replacement therapy in patients with Fabry's disease. J Int Med Res. 2007;35(4):574-81.
40. Siamopoulos KC. Fabry disease: kidney involvement and enzyme replacement therapy. Kidney Int. 2004;65(2):744-53.
41. Koeppe S, Neubauer H, Breunig F, Weidemann F, Wanner C, Sandstede J, et al. MR-based analysis of regional cardiac function in relation to cellular integrity in Fabry disease. International journal of cardiology. 2012;160(1):53-8.
42. Machann WB, F.;Weidemann, F.;Sandstede, J.;Hahn, D.;Kostler, H.;Neubauer, S.;Wanner, C.;Beer, M. Cardiac energy metabolism is disturbed in Fabry disease and improves with enzyme replacement therapy using recombinant human galactosidase A. Eur J Heart Fail. 2011;13(3):278-83.
43. Lenders M, Nordbeck P, Canaan-Kühl S, Kreul L, Duning T, Lorenz L, et al. Treatment switch in Fabry disease- a matter of dose? Journal of medical genetics. 2021;58(5):342-50.
44. Limgala RP, Jennelle T, Plassmeyer M, Boutin M, Lavoie P, Abaoui M, et al. Altered immune phenotypes in subjects with Fabry disease and responses to switching from agalsidase alfa to agalsidase beta. American journal of translational research. 2019;11(3):1683-96.
45. Politei J, Schenone AB, Cabrera G, Heguilen R, Szlago M. Fabry disease and enzyme replacement therapy in classic patients with same mutation: different formulations--different outcome? Clinical genetics. 2016;89(1):88-92.
46. Lin HYH, Y. H.;Liao, H. C.;Liu, H. C.;Hsu, T. R.;Shen, C. I.;Li, S. T.;Li, C. F.;Lee, L. H.;Lee, P. C.;Huang, C. K.;Chiang, C. C.;Lin, S. P.;Niu, D. M. Clinical observations on enzyme replacement therapy in patients with Fabry disease and the switch from agalsidase beta to agalsidase alfa. J Chin Med Assoc. 2014;77(4):190-7.
47. Ripeau D, Amartino H, Cedrolla M, Urtiaga L, Urdaneta B, Cano M, et al. Switch from agalsidase beta to agalsidase alfa in the enzyme replacement therapy of patients with Fabry disease in Latin America. Medicina (B Aires). 2017;77(3):173-9.
48. Vedder AC, Breunig F, Donker-Koopman WE, Mills K, Young E, Winchester B, et al. Treatment of Fabry disease with different dosing regimens of agalsidase: effects on antibody formation and GL-3. Molecular genetics and metabolism. 2008;94(3):319-25.
49. Pisani AS, L.;Visciano, B.;Capuano, I.;Sabbatini, M.;Riccio, E.;Messalli, G.;Imbriaco, M. Effects of switching from agalsidase Beta to agalsidase alfa in 10 patients with anderson-fabry disease. JIMD reports. 2013;9:41-8.
50. Tsuboi K, Yamamoto H. Clinical observation of patients with Fabry disease after switching from agalsidase beta (Fabrazyme) to agalsidase alfa (Replagal). Genetics in medicine : official journal of the American College of Medical Genetics. 2012;14(9):779-86.
51. Tsuboi KY, H. Clinical course of patients with Fabry disease who were switched from agalsidase-beta to agalsidase-alpha. Genetics in medicine : official journal of the American College of Medical Genetics. 2014;16(10):766-72.
52. Weidemann FK, J.;Duning, T.;Lenders, M.;Canaan-Kuhl, S.;Krebs, A.;Guerrero Gonzalez, H.;Sommer, C.;Uceyler, N.;Niemann, M.;Stork, S.;Schelleckes, M.;Reiermann, S.;Stypmann, J.;Brand, S. M.;Wanner, C.;Brand, E. Patients with Fabry disease after enzyme replacement therapy dose reduction versus treatment switch. Journal of the American Society of Nephrology : JASN. 2014;25(4):837-49.
53. Lenders MC-K, S.;Kramer, J.;Duning, T.;Reiermann, S.;Sommer, C.;Stypmann, J.;Blaschke, D.;Uceyler, N.;Hense, H. W.;Brand, S. M.;Wanner, C.;Weidemann, F.;Brand, E. Patients with Fabry Disease after Enzyme Replacement Therapy Dose Reduction and Switch-2-Year Follow-Up. Journal of the American Society of Nephrology : JASN. 2016;27(3):952-62.
54. Kramer J, Lenders M, Canaan-Kuhl S, Nordbeck P, Uceyler N, Blaschke D, et al. Fabry disease under enzyme replacement therapy-new insights in efficacy of different dosages. Nephrology, dialysis, transplantation : official publication of the European Dialysis and Transplant Association - European Renal Association. 2018;33(8):1362-72.
55. Germain DPW, F.;Abiose, A.;Patel, M. R.;Cizmarik, M.;Cole, J. A.;Beitner-Johnson, D.;Benistan, K.;Cabrera, G.;Charrow, J.;Kantola, I.;Linhart, A.;Nicholls, K.;Niemann, M.;Scott, C. R.;Sims, K.;Waldek, S.;Warnock, D. G.;Strotmann, J.;Fabry, Registry. Analysis of left ventricular mass in untreated men and in men treated with agalsidase-beta: data from the Fabry Registry. Genetics in medicine : official journal of the American College of Medical Genetics. 2013;15(12):958-65.

56. Weidemann F, Breunig F, Beer M, Sandstede J, Turschner O, Voelker W, et al. Improvement of cardiac function during enzyme replacement therapy in patients with Fabry disease: a prospective strain rate imaging study. Circulation. 2003;108(11):1299-301.
